# Stimulus symmetries can confound representational similarity analyses

**Published:** 2026-05-20

**Authors:** Farhad Pashakhanloo, Jacob A. Zavatone-Veth

**Affiliations:** 1 Center for Brain Science, Harvard University, Cambridge, MA, USA; 2 Society of Fellows, Harvard University, Cambridge, MA, USA

## Abstract

What can representational similarity matrices (RSMs) tell us about a neural code? As the popularity of these summary statistics grows, so too does the need for a more complete characterization of their properties. Here, we show that symmetries in network inputs can confound RSM-based analyses. Stimulus symmetries render many representations functionally equivalent, but these different configurations can lead to different RSMs. These different RSMs reflect qualitatively different representational geometries. We show that stochastic gradient descent or energetic regularization can generate sparse, drifting codes, leading in turn to drifting RSMs. Moreover, we demonstrate that these phenomena are present in networks trained to encode image data, where the symmetry is latent. Our results illustrate the challenges inherent in comparing nonlinear neural codes, when functionally-equivalent representations are not related by a simple rotation.

## 1 Introduction

The sensory world is replete with symmetries. Most famously, the identity of a visual object is invariant to its position and pose [1]. To robustly interpret the external world, the brain must grapple with these symmetries as it encodes and learns from natural data: the functionality of a representation should not be affected by globally transforming the stimulus space according to the symmetry.

In machine learning, recent years have seen significant interest in designing neural network architectures that are constrained to respect stimulus symmetries [2]. In particular, *equivariant* neural networks are engineered such that their internal representations are linear representations in the sense of group theory. That is, if one transforms the input according to the symmetry, the encodings at a given hidden layer of the stimulus before and after the transformation are related by a global linear transformation, most simply a rotation [2–4]. This property is advantageous mathematically, as it allows for decorrelation and disentangling as different symmetries act on the input [3, 5]. Moreover, it makes it easy to understand when two representations are related by the action of the stimulus symmetry. However, it is a strong constraint which is not inherently satisfied by most neural networks, whether natural or artificial.

How, then, should one analyze and understand neural representations of symmetric data? Perhaps most simply, given two representations—whether from different networks or from the same network at different stages of learning—how should one compare them? For both machine learning and for neuroscience, the search for principled measures of representational similarity is key to attempts to assess questions of convergence in encoding strategies, whether between artificial networks, between biological networks, or between brains and machines [6, 7].

Efforts to systematize this search have thus far focused mostly on designing metrics that are invariant to internal symmetries intrinsic to network architectures [6, 8, 9]. The motivation here is simple: two networks which are related by an intrinsic symmetry may erroneously appear different when viewed through the lens of a summary statistic that is not invariant to that symmetry. For example, one can compensate for global rotations of the encoding at one layer of a deep network by rotating the synaptic weights of the next layer. Thus, rotation-invariance is a reasonable desideratum for a summary statistic of deep representations. This invariance is realized by the matrix of dot-product similarities of encodings of a set of stimuli, known as the representational similarity matrix (RSM). Both as a precursor to downstream analyses—like representational similarity analysis (RSA) or centered kernel alignment (CKA)—or as an endpoint in itself, the RSM has gained broad popularity as a tool for understanding neural population codes [6, 9–14].

In contrast to the case of intrinsic symmetries, a systematic understanding of how to analyze representations of symmetric data is lacking. Concretely, outside of the setting of equivariant networks, previous works have not characterized how data symmetries might confound our attempts to understand neural representations. Most simply, one might ask whether RSM-based analyses could lead one to mistakenly identify two representations related by a stimulus symmetry as distinct, though they are functionally equivalent.

In this work, we show that stimulus symmetries can yield functionally equivalent representations that are not, in general, related by an orthogonal transformation in representation space, and therefore have distinct RSMs. This highlights a possible mismatch between function-preserving stimulus symmetries and the orthogonal transformations to which RSMs are invariant. As a tractable model setting, we focus on manifold-tiling neural codes, in which the neurons’ receptive fields form a regular lattice over stimulus space. The brain uses manifold-tiling codes to represent a range of physical and abstract variables, and they emerge through learning in a variety of model settings [15–17]. From a theoretical perspective, manifold-tiling representations are a simple example of a nonlinear neural code. For these codes, we show that the stimulus symmetry does not in general act as a rotation on the representation space, meaning that examining the RSM would lead one to believe that codes related by the symmetry are distinct. We illustrate this through a variety of controlled experiments, spanning synthetic and image data. Together, our results show how biologically-plausible nonlinear neural codes can be challenging to compare in the presence of stimulus symmetries.

## 2 Gauge-dependent RSMs in a toy model

**Figure 1:**
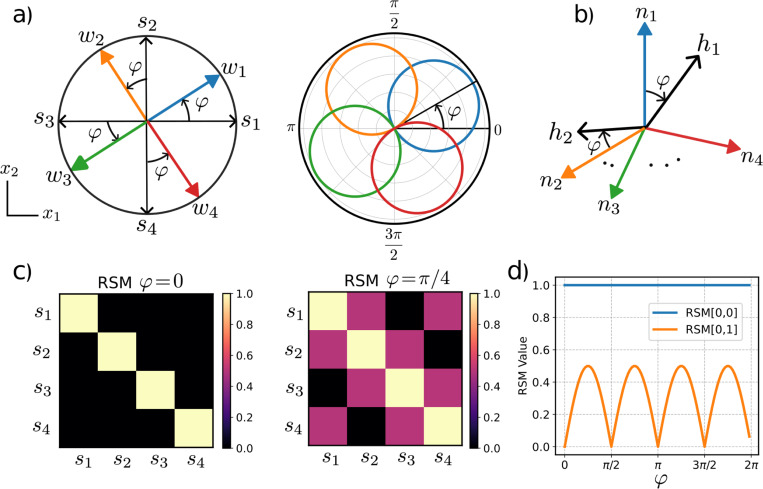
Gauge-dependent representations in a toy model. a) Left: RF center vectors ($w_{i}$) of four neurons tiling a one-dimensional ring. To specify a tiling, one must choose a global orientation φ. Right: corresponding angular tuning curves. b) Representations $h_{1}$ and $h_{2}$ of two trial stimuli $s_{1}$ and $s_{2}$ in the four-dimensional representation space. The angle between $h_{1}$ and $h_{2}$ depends on φ. c) RSMs for two values of the gauge variable φ. d) RSM diagonal and off-diagonal entries as a function of φ. The variation of the RSM with φ is the key phenomenon which this work explores.

We begin with a toy example that illustrates the key phenomenon, motivated by classic models for orientation encoding in neuroscience [11]. Consider a setting where data lies on a one-dimensional ring ($x\in S^{1}$), encoded by n neurons with localized receptive fields that tile this space uniformly. Considering this tiled representation, we see there is an ambiguity: given a particular spacing between receptive fields, there exist many possible tilings of the stimulus manifold which are functionally equivalent given the rotational symmetry. Therefore, to completely specify the representation, one must fix a gauge; one must anchor the lattice of receptive fields (Fig. 1a).^1^

Concretely, the activation of the i-th neuron in response to an input x is

(1) $$h_{i}(x)=ReLU\left(w_{i}^{⊤}x\right),\;\text{where}\;w_{i}=\left(cos\;\theta_{i},sin\;\theta_{i}\right)$$

is the (unit-length) weight vector associated with that neuron, defined by an angle

(2) $$\theta_{i}=2(i-1)\pi /n+\varphi \;\text{for}\;i\in [n].$$

Here, φ is an arbitrary offset angle which defines the global configuration; it is the gauge variable. Since each neuron has a receptive field (RF) that covers half the space (π radians on the ring), n=4 is the minimum number of neurons that can cover this space faithfully (Fig. 1a; see App. C). To be precise, for any φ, one can perfectly recover $x\in S^{1}$ from h(x) using a linear readout whose weights are rotated to compensate for the change in the representation as φ varies (App. A.1).

In this configuration, one can track the representations of a set of trial stimuli, $s_{1}=(1,\;0{)}^{⊤}$, $s_{2}=(0,\;1{)}^{⊤}$, $s_{3}=(-1,\;0{)}^{⊤}$ and $s_{4}=(0,-1{)}^{⊤}$.^2^ Collecting the representations into a matrix $H=\left[h\left(s_{1}\right),h\left(s_{2}\right),h\left(s_{3}\right),h\left(s_{4}\right)\right]$ and defining the associated $RSM=H^{⊤}H$, we have

(3) $$H=\left(\begin{array}{cccc} cos(\varphi ) & sin(\varphi ) & 0 & 0\\ 0 & cos(\varphi ) & sin(\varphi ) & 0\\ 0 & 0 & cos(\varphi ) & sin(\varphi )\\ sin(\varphi ) & 0 & 0 & cos(\varphi ) \end{array}\right)\;\text{and}\;RSM=\left(\begin{array}{cccc} 1 & \rho (\varphi ) & 0 & \rho (\varphi )\\ \rho (\varphi ) & 1 & \rho (\varphi ) & 0\\ 0 & \rho (\varphi ) & 1 & \rho (\varphi )\\ \rho (\varphi ) & 0 & \rho (\varphi ) & 1 \end{array}\right)$$

for φ∈[0,π/2), where ρ(φ)=sinφcosφ. Increasing φ beyond π/2 is equivalent to circularly permuting the neuron labels and taking the residue of φ modulo π/2. Thus, the RSM is clearly gauge-dependent (Fig. 1c-d). This change is qualitatively substantial, as by changing φ one can achieve either a purely diagonal RSM or one where the off-diagonal elements are as large as one-half.

Therefore, in a simple case where changing the gauge variable φ corresponds to an overall rotation of the tuning curves, the RSM depends on the choice of gauge. This results from the fact that the global rotation of the tuning curves does not translate to an overall rotation in the representation space; the angle between the representations of two stimuli can change (Fig. 1b).

## 3 Formalizing RSM gauge-dependence for general data symmetries

The example in §2 is a simple instance of a more general phenomenon, which we now formalize. We first formalize the notions of symmetry in the data and functional equivalence for representations, and finally state the gauge dependence of the RSM. For ease of exposition, we will consider a highly idealized setup, but these assumptions could in principle be relaxed. We emphasize that this section is not essential for understanding the rest of the paper; the reader can skip to Section 4.

### 3.1 Symmetries in the latent space

In general, we allow the data symmetries to act on a latent variable rather than on the observations themselves. Concretely, suppose we have a d-dimensional latent space Z upon which a compact group G acts, via · : G×Z→Z. The symmetry of the data is manifested in two ways: First, there is a G-invariant metric $d_{Z}$ on Z, *i.e.*, a metric such that $d_{Z}\left(g\cdot z,g\cdot z^{'}\right)=d_{Z}\left(z,z^{'}\right)$ for all $z,z^{'}\in Z$ and all g∈G. Second, the latent variables are distributed according to a G-invariant probability measure $\mu_{Z}$, *i.e.*, a measure such that $\mu_{Z}(g\cdot A)=\mu_{Z}(A)$ for all A⊆Z, and that $\mu_{Z}(Z)=1$.

Given this symmetric latent space, we suppose that we observe m-dimensional data x∈X (where m≥d). We allow the observations to depend both on the sampled latent variable z∈Z and on some non-symmetric nuisance degrees of freedom ξ∈Ξ through a function χ:Z×Ξ→X such that x=x(z,ξ)=χ(z,ξ). We assume that the nuisance degrees of freedom ξ are distributed according to some probability measure $\mu_{\Xi }$, and are statistically independent of the latent variables z. For simplicity, we will assume that χ is invertible almost everywhere, such that we can uniquely recover z and ξ from x. In the toy setting introduced in §2, we have no nuisance variables, and X≃Z.

We then define an action of the symmetry group on the data space by its action on the latent space Z:

(4) $$g\cdot x=x\left(g\cdot z,\xi \right).$$

Our ability to use this definition for the action of G on the whole of X depends on the data-generating function χ being invertible. Moreover, we suppose that we have a metric $d_{X}$ on X that is G-invariant, *i.e.*, $d_{X}\left(g\cdot x,g\cdot x^{'}\right)=d_{X}\left(x,x^{'}\right)$ for all $x,x^{'}\in X$ and all g∈G. This assumption is restrictive, but it holds at least approximately for some realistic settings of interest (see §7). For example, when considering a pair of images where one is a rotated version of another, the distance should depend on their relative angles.

### 3.2 Gauge invariance of decoding accuracy

We now consider encoding the data x∈X by a deterministic encoding function $h:X\to H\subseteq R^{n}$. With autoencoding in mind—though our ideas could be extended to other settings—we assess the quality of the encoding based on how well the data can be decoded. That is, we seek a decoding function f:H→X such that $d_{X}(x,f(h(x)))$ is small, say on average over the distribution of x. We therefore define the average error incurred by encoding x with h and decoding with f:

(5) $$E[h,f]=E_{z∼\mu_{Z},\xi ∼\mu_{\Xi }}\left[d_{X}(x(z,\xi ),f(h(x(z,\xi ))))\right].$$

We can define the action of the symmetry group on the encoding by its action on the inputs:

(6) $$(g\cdot h)(x)=h\left(g^{-1}\cdot x\right)=h\left(x\left(g^{-1}\cdot z,\xi \right)\right).$$

This transformation is functionally irrelevant, as the data can be decoded equally well from either h or $h^{'}\equiv g\cdot h$. We therefore refer to it as a *gauge transformation*. In particular, defining the action of G on the decoder by viewing the decoded example f(a) as an element of X (*i.e.*, (g⋅f)(a)=g⋅f(a) for any a∈H), we have

(7) E[g⋅h,g⋅f]=E[h,f]

for all g∈G as a consequence of the G-invariance of $d_{X}$ and of $\mu_{Z}$ (see App. B).

This constructively proves the existence of a decoder $f^{'}=g\cdot f$ from $h^{'}=g\cdot h$ that achieves accuracy equal to that resulting from decoding from h using f. Our restrictive assumptions—in particular the invertibility of the mapping between latents and data and the G-invariance of $d_{X}$—make this equal-accuracy construction straightforward. These conditions could be weakened if one wants only for the accuracies to be within some tolerance of one another. Then, f and $f^{'}$ may not be related simply by a gauge transformation, making it harder to prove the existence of a suitable $f^{'}$.

### 3.3 Gauge dependence of the RSM

Though the decoding accuracy is gauge-invariant, the encodings themselves are clearly not in general gauge-invariant. This gauge-dependence will in general carry over to the RSM, as it is invariant only if the gauge transformation preserves pairwise inner products in representation space. Formally, the RSM is defined for $x,x^{'}\in X$ by the Euclidean inner product of h(x) and $h\left(x^{'}\right)$:

(8) $$RSM_{h}\left(x,x^{'}\right)=h(x{)}^{⊤}h\left(x^{'}\right)=\sum_{i=1}^{n}h_{i}(x)h_{i}\left(x^{'}\right).$$

For the RSM to be gauge-invariant, we must have

(9) $$RSM_{g\cdot h}\left(x,x^{'}\right)=RSM_{h}\left(x,x^{'}\right)$$

for all $x,x^{'}\in X$ and any g∈G. As we show formally in Appendix B, this holds if and only if there exists an orthogonal matrix O∈O(n), depending on the group element g but not on x, such that

(10) $$\left(g\cdot h\right)\left(x\right)=O\left(g\right)h\left(x\right).$$

This result follows from the classical fact that two sets of vectors having the same Gram matrix must be related by an orthogonal transformation [19].

**Figure 2:**
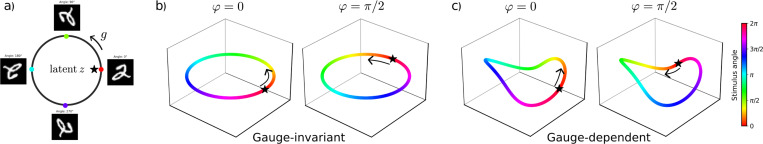
Gauge dependence and the geometry of representational manifolds. The schematics show a) the latent space $Z=S^{1}$, and two examples of b) linear and c) nonlinear embeddings of it in $R^{3}$. Points are color-coded by the stimulus angle, and angle zero is marked by star (⋆). The left and right columns have a gauge difference of Δφ=π/2. The two manifolds in (b) can be transformed into each other using an orthogonal transformation, but this is not possible in (c). Consequently, per Section 3.3, the representations in (c) have gauge-dependent RSMs.

In group-theoretic terms, this would mean that the encoding is an orthogonal linear representation of G [20]. This is satisfied by construction for equivariant architectures [2–4]. However, as we saw in §2—and as we will see in the subsequent sections of the paper—this is not in general the case, even for simple and biologically-reasonable nonlinear encodings. We illustrate the geometry underlying the gauge-variance of the RSM in Figure 2: if the encoding is nonlinear, then the representational manifolds before and after a gauge transformation are not related by a rotation.

The analysis above considers invariance at the level of the full RSM kernel, *i.e.*, for *any* pair of stimuli $x,x^{'}\in X$. In practice, one can test only a finite set of trial stimuli, as we did for the toy model in (3) (though, see Fig. S1). Formally, showing gauge dependence of the RSM for a finite set of trial stimuli is sufficient to show that the representation is not orthogonally equivariant. However, equality of the finite RSMs is not in general sufficient to show orthogonal equivariance on the full stimulus space (see Appendix B). In principle, the choice of trial stimuli could either mask or exaggerate the gauge-dependence of the full RSM.

### 3.4 Suppressing irrelevant variables: receptive fields

Studying the RSM as a function of the data x is in practice inconvenient for two reasons: First, the latents z may be of significantly lower dimension than x. Second, it depends on the nuisance variable ξ, about which we may not care. In keeping with neuroscience parlance and practice [11, 14, 15], we isolate the z-dependence of the encoding by defining the *receptive fields* $r:Z\mapsto R^{n}$. Then, we can consider instead the receptive field RSM: $RSM_{r}\left(z,z^{'}\right)=r(z{)}^{⊤}r\left(z^{'}\right)$ for $z,z^{'}\in Z$. Like $RSM_{h}$, $RSM_{r}$ is in general not gauge-invariant. This complication is not present if X≃Z, as in the toy model of §2 and some of the examples we construct below.

How the receptive fields should be defined depends on the nature of the irrelevant variables. One simple approach is to average over the nuisance variables, and define $r(z)=E_{\xi }[h(x)]$. For instance, if one encodes data x drawn from a spherically-symmetric distribution using a ReLU encoder h(x)=ReLU(Wx)—as we will in subsequent sections—we can isolate the symmetric latent $z\in S^{d-1}$ by averaging. Concretely, writing x=ξz for $\xi =\|x{\|}_{2}$, we have $r(z)=E_{\xi }[h(z,\xi )]=E[\xi ]\;ReLU(Wz)$, and the radial variability drops out. Another possibility would be to consider the responses for varying z given a fixed reference value for ξ.

## 4 Towards real data: factors influencing gauge dependence in the toy model

In §2, we showed gauge-dependence of the RSM in a highly idealized toy model. With the objective of demonstrating that this phenomenon is present in networks where the RFs are learned rather than hand-designed in mind, we build intuition by considering how introducing various departures from this toy model affect the gauge-dependence of the RSM. Specifically, we will study the effects of the number, the width, and the amplitude of the RFs.

We quantify the φ-dependence of the RSM by:

(11) $$\Delta :={max}_{\varphi }\left(RSM_{off}\right)-{min}_{\varphi }\left(RSM_{off}\right),$$

where $RSM_{off}$ is an off-diagonal element of the normalized RSM.^3^ The n=4 case in §2 corresponds to the minimum number of ReLU neurons needed to tile the ring. In this case, the tuning width of each neuron is π, as the RF covers a half-plane. We systematically varied the number and tuning width of RFs by adjusting the weight magnitude and bias of each ReLU neuron (see App. E.1 for details), and calculated Δ correspondingly. In general, Δ depends on the choice of trial stimuli. Unless otherwise stated, we calculate Δ for stimuli that are separated by half of the tuning width, which is equivalent to the maximum Δ among all possible trial stimuli.

**Figure 3:**
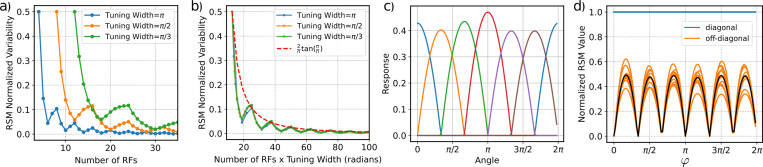
Factors influencing RSM gauge dependence. a,b) RSM variability (Δ) as a function of number of RFs and tuning width. In (b), the dashed red line shows the prediction from Eqn 12. c) RFs with amplitude noise (additive Gaussian noise with standard deviation 0.1). d) Corresponding φ-dependence of RSM over 100 realizations of noisy RFs (black line: average).

For a given tuning width, increasing the number of RFs suppresses variability in the RSM (Fig. 3a-b). However, the product of tuning width and the number of RFs matters in this relationship: for small tuning widths, the variability Δ is non-negligible even if the number of RFs is large (Fig. 3a-b and S4). In Appendix C, we show that for n a multiple of 4 and a tuning width of π, the RSM has a simple closed-form expression which generalizes that found for n=4; all that changes is the constant value of the diagonal elements and the details of the function ρ(φ). This leads to:

(12) $$\Delta =\frac{2}{n}tan\;\left(\frac{\pi }{n}\right),$$

which holds for n a multiple of 4. As n→∞, the variability in the RSM vanishes as $O\left(n^{-2}\right)$. This is plotted as a dashed red line in Fig. 3b. Changing the width of the RFs is approximately equivalent to re-scaling the input (App. E.1), which explains the near-collapse of curves in Fig. 3b.

Finally, we studied the effect of noisy RFs on the RSM variability. Fig. 3c,d show an example of RFs with amplitude noise and the corresponding gauge dependence curves for multiple runs. Despite the noise, the expected modulation by φ is noticeable, especially in the average (black curve – additional results are shown in Fig. S5).

## 5 Learning-induced manifold-tiling and drift in representations of the circle

So far, we showed that a manifold-tiling solution can create a varying RSM as a function of the functionally-irrelevant gauge variable, provided that the tiling is not too dense. Here, we study this in a learning scenario. This includes a two-layer autoencoder with ReLU neurons that is trained under SGD and weight decay with isotropic d-dimensional Gaussian data. We give more details and additional experimental results in Appendix E.

### 5.1 SGD need not select a unique RSM

As the first example, we replicate our toy model results with SGD. We find that SGD training replicates the uniform-tiling solution for the n=4 neurons in the hidden layer. Over the course of continual training, the tuning curves and the RSM drift over time, without selecting a particular solution (Fig. S6). This ambivalence also holds across different realizations of the learning started from different initializations of the weights (Fig. S7).

### 5.2 SGD inductive bias or energetic regularization can generate sparsely-tiling solutions

**Figure 4:**
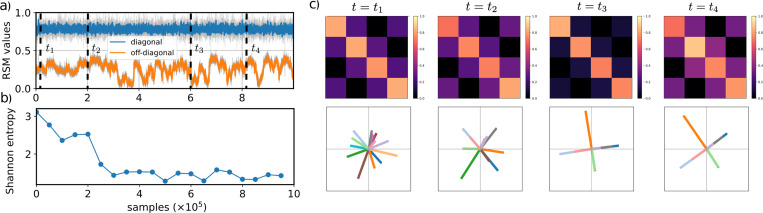
Collapse of neurons and RSM drift under continual SGD training. a) RSM values over time. b) Entropy of the distribution of cosine of pairwise angles between all neurons’ weights. c) RSMs (top), and the corresponding weight vectors (bottom) at different snapshots during training. Alignment of neurons’ weights (n=15) into 4 orthogonal directions is evident through learning.

**Figure 5:**
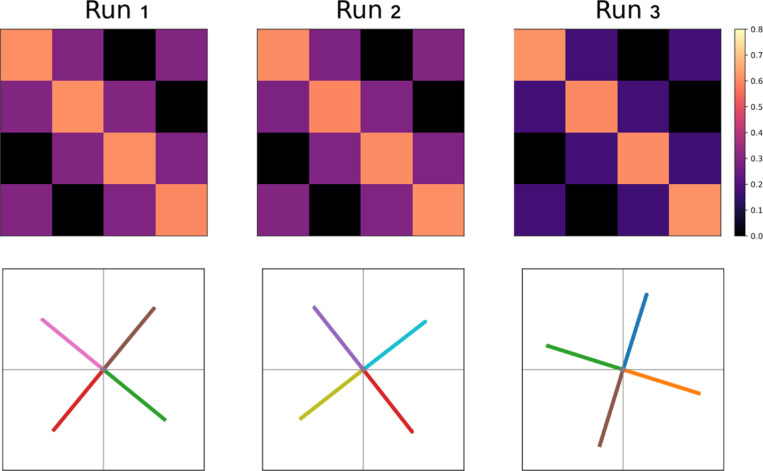
Gauge dependence of RSM under an $L_{1}$ penalty on activations. RSM plots (top) and neurons’ weight vectors (bottom) for three different runs (n=15 neurons). Similar to Figure S8, the batch size is large but the $L_{1}$ penalty on the hidden-layer activations leads to all but 4 neurons remaining active. Each simulation is run with 5 × 10^4^ samples seen (batch size of 100).

We found in §4 that for a fixed tuning width, densely tiling the manifold with many neurons suppresses variability in the RSM. Here, we demonstrate two ways that learning can lead to solutions with highly-variable RSMs, even when the number of hidden neurons is large. First, SGD can align the receptive fields of multiple neurons, leading to a solution with *effectively* only four neurons (Fig. 4). After this collapse, the gauge continues to drift, and the RSM varies accordingly (see panels $t=t_{3}$ and $t=t_{4}$ in Fig. 4c). This collapse can occur when SGD noise is large; see Fig. S8 for an example of a low-noise regime. Second, regularizing the activations with an $L_{1}$ penalty—to mimic energetic costs—can lead to all but four neurons becoming silent (see Fig. 5). This effect also allows RSM variability to exist despite a high number of neurons. Due to the fact that data are sampled uniformly from the latent space, the $L_{1}$ penalty does not necessarily fix the gauge (App. C.5).

## 6 Structured RSM variability for higher-dimensional spherical stimuli

We have thus far considered a two-dimensional stimulus with a one-dimensional symmetry group (rotations in the plane). However, in keeping with the general formalism of §3, the same gauge redundancy is present in representations of higher-dimensional symmetric manifolds. Analytical study of tiled representations of higher-dimensional manifolds is challenging because the maximally uniform arrangement of receptive field centers is generally unknown, even for the sphere $S^{2}$ [21]. However, if we tile the hypersphere $S^{d-1}$ with a reflection-symmetric arrangement of neurons—grouped in pairs with oppositely-oriented receptive fields—we can predict the structure of variability in the RSM induced by the d(d-1)/2-dimensional continuous SO(d) gauge symmetry.

**Figure 6:**
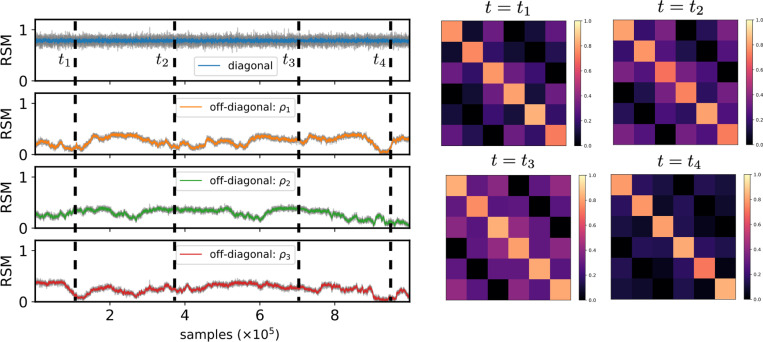
RSM variability as a result of continual training for three-dimensional input. (Left) Components of the empirical RSM as a function of time. The non-zero off-diagonal elements are grouped into three distinct sets ($\rho_{1}$, $\rho_{2}$, and $\rho_{3}$) based on the predictions derived in Appendix D (this implies entries [0,1],[0,4],[1,3], [3,4], and their transpose vary as $\rho_{1}$, and similarly for other groups). Gray curves correspond to the group members and the colored curves denote the group averages. (Right) RSM matrices at different snapshots demonstrate variability through time. There are n=6 ReLU neurons in the network.

Concretely, we consider an encoding $h(x)=ReLU\left(W^{⊤}x\right)$, where the weight matrix $W=(U^{⊤},-U^{⊤})$ for some $U\in R^{n/2\times d}$. Now, the test stimuli are the d cardinal basis vectors and their negations. To perfectly recover the test stimuli, the matrix U must be semi-orthogonal, *i.e.*, $U^{⊤}U=I_{d}$. For any such semi-orthogonal U, the RSM evaluated on the test stimuli is given by

(13) $$RSM=\left(\begin{array}{cc} I_{d}+R & R\\ R & I_{d}+R \end{array}\right),$$

where R is a d×d symmetric matrix with zeros along the diagonal, which thus has d(d-1)/2 independent elements (see derivation in App. D). In three dimensions, there are therefore three sets of distinct non-zero off-diagonal elements of the RSM, each of which varies over time during continual training (Fig. 6). Not only is the RSM highly structured in the sense that its elements cluster into groups, but the variability of these groups is correlated (see also Fig. S9 for d=10, where there are d(d-1)/2=45 distinct groups). In the simulations, we use isotropic Gaussian data, meaning that they include a radial nuisance variable.

## 7 RSM variability with latent symmetries in image data

**Figure 7:**
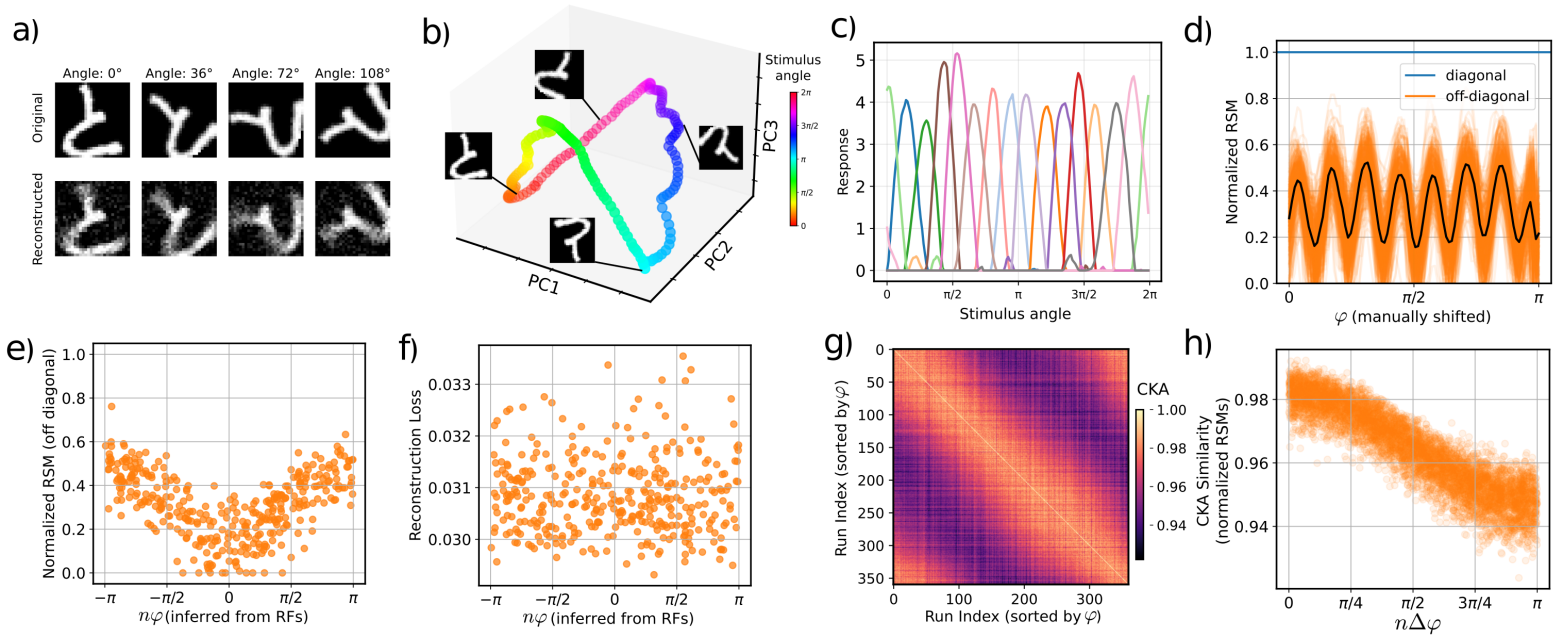
RSM variability in autoencoding a rotated image manifold. Autoencoders were trained on rotated versions of a digit from Kuzushiji-MNIST dataset compiled by Clanuwat et al. [22]. a) Four examples of the original and reconstructed images in an instance of the trained model. b) 3D PCA of the hidden layer activations in a trained model in response to all stimuli. Each point is color-coded by the rotation angle of the stimulus. c) RFs in a model with n=15 active neurons. d) φ−dependence curves of RSM. Each orange curve corresponds to a run and is a result of manually shifting the RFs. Curves are aligned and the black curve shows the average. e) Scatter plot of normalized off-diagonal RSM vs. the RF-inferred φ across different trained networks. Each point shows a different run of the model that had n=15 active RFs (see Fig. S11 for different n’s). The off-diagonal RSMs are calculated for two stimuli that are α=25^·^ apart. f) Same as (e) but the y-axis is the reconstruction loss for each run. g) CKA similarity matrix calculated between normalized RSMs of different runs. The runs are sorted based on the inferred φ. h) Scatter of CKA similarity as a function of gauge difference (each point represents a pair of runs, and for visualization purposes only 1/10 of the data are shown).

So far, we have demonstrated RSM variability in networks trained on Gaussian data. Here, we consider a more realistic case where the data are generated by rotations of a static image from the Kuzushiji-MNIST dataset [22]. The latent space is $S^{1}$, corresponding to stimuli with different rotation angles, but the stimulus space is more complicated than previous cases. We train a two-layer autoencoder with ReLU nonlinearity on 180 rotated versions of the image (Fig. 7a; see App. E.2.2 for model details). Localized and noisy RFs are formed in the hidden layer, and they approximately tile the $S^{1}$ latent space (Fig. 7b,c).

To study the gauge dependence of RSM, we create 1000 instantiations of the trained model and perform the following two analyses. First, similar to the previous φ-dependence curves in the toy models (e.g. in Fig. 3d), for each trained network we manually change the gauge by circularly shifting the RFs and compute corresponding RSMs. As shown in Fig. 7d, the φ-dependence curves oscillate at intervals of 2π/n, consistent with the toy model results. Second, since the models are trained independently, we expect them to have randomly distributed gauge φ. We utilize this fact, and for each run infer φ by Fourier-transforming the envelope of the RF curves, and extracting the phase associated with the dominant frequency (see App. E.2.2 and Fig. S10). We then plot a scatter of the normalized off-diagonal element of the RSM as a function of this inferred φ (Fig. 7e). The U-shape visible in this plot confirms the expected gauge dependence of the RSM across the models (Pearson’s r=0.76, p<0.001, between the RSM and absolute phase). Importantly, the reconstruction loss shows no clear φ-dependence (Fig. 7f, Pearson’s r=0.03, p=0.56; see additional results in Fig. S11). Finally, we show that the CKA similarity drops significantly as a function of the difference in the gauge variable (Fig. 7g-h, Pearson’s r=-0.91, p<0.001). Overall, these results show that, in a more realistic setup with noisy RFs, the gauge dependence of the RSM can be distinguished from other sources of RSM variability.

As a final illustration of how latent rotational symmetries in image data can affect RSM-based analyses, we consider several standard pretrained general-purpose image models [23–25]. Previous works have shown that image models not constrained to obey rotation-equivariance generally do not learn this symmetry from data [3, 26–28]. Consistent with this finding, we show in Figure S13 that representations of rotated versions of the same image trace out non-trivial manifolds, and that their RSMs vary systematically with relative rotation angle, as measured by CKA. This experiment does not show that these representations are functionally equivalent in the same sense as the autoencoders considered above, but it illustrates that standard general-purpose vision models need not render latent stimulus rotations invisible to RSM-based model comparisons.

## 8 Discussion

Representational similarity measures have been used extensively in neuroscience and ML to compare internal representations of artificial and biological networks [6, 10, 13, 29, 30]. In this work, we demonstrate in simple settings that such measures may not be invariant under transformations that respect symmetries in the data. Specifically, we showed that functionally equivalent arrangements of receptive fields can lead to qualitatively different RSMs.

Theoretical work on the statistical physics of neural networks suggests that various summary statistics—including RSMs—may be stable when the number of neurons in each hidden layer of the network tends to infinity [30]. Our results on when the RSM variability tends to zero with the number of neurons in §4 are compatible with those works. However, we showed that there are regimes in which learning may lead to solutions with a low *effective* number of neurons. In evaluating how high the effective neuron count must be in order to suppress variability in the RSM, one must consider the intrinsic dimensionality of the symmetric stimulus manifold. From the curse of dimensionality, we expect the number of neurons required to suppress variability in the RSM should scale exponentially with the intrinsic dimension of the manifold.

Ongoing drift in representations despite stable task performance has been observed in the brain and in artificial neural networks [31, 32]. Previous works have sought to explain this phenomenon based on internal symmetries, yielding models with changing representations but stable RSMs [32–35]. Complementing these results, our work shows how extrinsic stimulus symmetries can also lead to drifting representations over learning, with RSMs that change over time. Note that changing RSMs can occur even in networks that explicitly or implicitly impose a loss to align the RSMs of the stimuli and the representations (*e.g.*, similarity matching networks as in [16, 33, 35]). This is because symmetry along the latent dimension can allow RSMs to vary, while keeping the similarity matching objective fixed (see Fig. S3 and App. B.3). Additionally, there is experimental evidence showing changing RSMs over time in cortex [32, 36]. Our results here show that, to reliably interpret the functional significance of such observations, one must take into account (potentially unknown) symmetries in data.

A growing body of work aims to characterize the conditions under which networks have unique optimal representations. From a neuroscience perspective, a key question is when biologically-inspired constraints—like non-negativity of neural activity or energy constraints that can be modeled by regularization—select a unique RSM [37–43]. These works, more broadly, seek to determine when axis-aligned solutions, in which each hidden neuron in an autoencoder responds only to a single latent dimension, are optimal [38, 39]. However, how symmetries in data affect these results remains unknown. Indeed, in the presence of symmetries, there may be no preferred basis for the latent stimulus, so the very notion of axis-alignment can become ill-defined. Here, we showed that in the presence of continuous data symmetries, the constraints of energy minimization (*e.g.* in the form of an $L_{1}$ penalty on neural activity) and non-negativity may not be sufficient to select a unique RSM.

In the representations we study, the underlying reason why the RSM is not gauge-invariant is that solutions with different gauge angle are not related by a simple rotation in representation space (Fig. 2). We have studied manifold-tiling neural codes as a test case for this phenomenon, as they provide a minimal, neuroscience-inspired example of a nonlinear neural code. However, the idea that data symmetries can lead one to erroneously recognize two functionally equivalent representations as being distinct generalizes beyond the manifold-tiling setting, because many representations are not constrained to be orthogonally equivariant. To compare representations in a way that is invariant to data symmetry, one must design a metric that is invariant to the symmetry transformations, which in general requires knowledge of the stimulus space. Recent work on comparing representations using the pullback metric on the intrinsic data manifold could potentially address this problem [44, 45], but such methods require complete knowledge of the stimulus distribution. Absent such strong priors, it is unclear how to account for the gauge-invariance of representations. Our study motivates future attempts at seeking such metrics.

## Supplementary Material

Supplement 1

## Footnotes

1 Our use of the term “gauge” is inspired by physics, though the phenomenon we describe is not identical, as here the gauge is observable but not functionally relevant [18].

2 We use these trial stimuli to show a compact form of the RSM, but the data is distributed uniformly over all of $x\in S^{1}$. See App. A.2 and Fig. S1 for the RSM for arbitrary stimuli in $S^{1}$.

3 The normalized RSM, or cosine similarity matrix, is given by normalizing the off-diagonal elements by the corresponding diagonal entries. Normalization does not remove the φ-dependence.

## Code Availability

The codes and functions to reproduce the main results of this paper are publicly available at: https://github.com/fpashakhanloo/stimulus-sym-rsa.
