# Supplementary material for: Stimulus symmetries can confound representational similarity analyses
Source: ArXiv. 2026 May 20:arXiv:2605.21324v1. Preprint. [Version 1] (PMC13229085)
Supplement: Supplement 1 [file NIHPP2605.21324v1-supplement-1.pdf]

Contents

A Additional results for toy model with $n = 4$ neurons **16**

A.1 Readout . . . 16

A.2 RSM for arbitrary angles . . . 16

A.3 Action of the gauge group on the four-neuron representation of the circle . . . 16

A.4 Alignment between two RSMs . . . 17

B Deferred derivations related to general formalism **18**

B.1 Gauge-invariance of decoding accuracy . . . 18

B.2 Conditions for gauge-invariance of the RSM . . . 18

B.3 Similarity matching between the stimulus and the representation . . . 20

C Detailed analysis of the toy orientation tuning model with many neurons **21**

C.1 Reconstruction . . . 21

C.2 Evaluation of the similarity matrix elements . . . 22

C.3 Large-n limit . . . 23

C.4 Normalized variability . . . 23

C.5 Penalizing the L_1 norm of activations does not fix a gauge . . . 24

C.6 The RSM for arbitrary angular differences . . . 25

C.7 Amplitude variability . . . 29

D Reflection-symmetric tilings of the sphere in higher dimensions **30**

D.1 Structure of the reconstruction . . . 31

D.2 Structure of the RSM . . . 31

E Additional experimental details **32**

E.1 Toy setup . . . 32

E.2 Neural network simulations . . . 33

E.3 Pretrained vision models (Figure S13) . . . 33

F Additional figures **35**

A Additional results for toy model with $n = 4$ neurons

A.1 Readout

In our two-layer toy model, the gauge symmetry rotated the first layer weights. For completeness, here we show that a similar transformation applied to the readout weights leaves the input-to-output mapping and hence the loss unchanged. If $W \in \mathbb{R}^{n\times 2}$ and $U \in \mathbb{R}^{2\times n}$ are the first and second layer weights of the network, it is easy to check that for $n = 4$, the solution satisfies: $U = W^T$. This essentially means that the outgoing weights of each neuron, u_i, tracks the incoming weights w_i, to have: $u_i = [\cos(\theta_i), \sin(\theta_i)]$ for $\theta_i = 2(i-1)\pi/n + \varphi$. To verify this, take the arbitrary stimulus $s_\beta = [\sin(\beta), \cos(\beta)]^T$, where $-\varphi \leq \beta < \pi/2 - \varphi$. The output for this stimulus is:

$$y_\beta = Uh_\beta = \begin{pmatrix} \cos(\theta_1) & \cos(\theta_2) & \cos(\theta_3) & \cos(\theta_4) \\ \sin(\theta_1) & \sin(\theta_2) & \sin(\theta_3) & \sin(\theta_4) \end{pmatrix} \begin{pmatrix} \sin(\varphi+\beta) \\ \cos(\varphi+\beta) \\ 0 \\ 0 \end{pmatrix} \tag{14}$$

$$= \begin{pmatrix} \cos(\varphi)\sin(\varphi+\beta) - \sin(\varphi)\cos(\varphi+\beta) \\ \sin(\varphi)\sin(\varphi+\beta) + \cos(\varphi)\cos(\varphi+\beta) \end{pmatrix} \tag{15}$$

$$= \begin{pmatrix} \sin(\beta) \\ \cos(\beta) \end{pmatrix} \tag{16}$$

$$= s_\beta. \tag{17}$$

By symmetry, this shows that gauge transformations do not change the network function for any stimulus or gauge angle. See also Appendix D.1 for a more general analysis of reconstruction that covers this special case.

A.2 RSM for arbitrary angles

Previously, we considered orthogonal stimuli $s_1 = [1, 0]^T$, $s_2 = [0, 1]^T$, etc. for calculating RSM. Here, we calculate RSMs for arbitrary angles. Consider the following stimuli:

$$s_\alpha = [\cos(\alpha), \sin(\alpha)]^T, \quad s_\beta = [\sin(\beta), \cos(\beta)]^T, \tag{18}$$

for $-\pi/2 + \varphi \leq \alpha \leq \varphi$ and $-\varphi \leq \beta < \pi/2 - \varphi$. Here, α and β denote deviations from s_1 and s_2 respectively, and by symmetry this covers any arbitrary angle on the circle. The representations of stimuli s_1, s_α and s_β are as follows:

$$h_1 = \begin{pmatrix} \cos(\varphi) \\ 0 \\ 0 \\ \sin(\varphi) \end{pmatrix}, \quad h_\alpha = \begin{pmatrix} \cos(\varphi-\alpha) \\ 0 \\ 0 \\ \sin(\varphi-\alpha) \end{pmatrix}. \quad h_\beta = \begin{pmatrix} \sin(\varphi+\beta) \\ \cos(\varphi+\beta) \\ 0 \\ 0 \end{pmatrix}. \tag{19}$$

(20)

This leads to the following RSM entries:

$$RSM_{s_1,s_\alpha} = \cos(\varphi)\cos(\varphi-\alpha) + \sin(\varphi)\sin(\varphi-\alpha) = \cos(\alpha) \tag{21}$$

$$RSM_{s_1,s_\beta} = \cos(\varphi)\sin(\varphi+\beta) \tag{22}$$

The first RSM does not depend on the gauge variable. This is a case where the set of activated neurons between two stimuli are identical. The second RSM depends on φ. Note that, in general, even if two stimuli are nearby, there could be a range of φ for which the set of activated neurons are different. This leads to a normalized variability (Δ) that varies as function of angles between two stimuli. See Fig S1b for such a curve. For the generalization of these results to many neurons, see Appendix C.6.

A.3 Action of the gauge group on the four-neuron representation of the circle

One might ask what the concrete form of the action of the $\mathrm{SO}(2)$ gauge group on the four-neuron representation of the circle is. Consider the representation of an arbitrary angle θ:

$$h(\theta) = \begin{pmatrix} \mathrm{ReLU}(\cos\theta) \\ \mathrm{ReLU}(\sin\theta) \\ \mathrm{ReLU}(-\cos\theta) \\ \mathrm{ReLU}(-\sin\theta) \end{pmatrix} \tag{23}$$

The gauge group acts via

$$g_\varphi \cdot h(\theta) = h(\theta - \varphi). \tag{24}$$

It is clear that this is a piecewise-linear action on the representation, and one can see that there are 16 distinct linear regions corresponding to distinct activation patterns before and after the gauge transformation. For instance, suppose that we are in the region

$$\theta \in [0, \pi/2], \varphi \in [0, \theta) \cup [3\pi/2 + \theta, 2\pi) \tag{25}$$

such that

$$h(\theta) = \begin{pmatrix} \cos\theta \\ \sin\theta \\ 0 \\ 0 \end{pmatrix} \quad \text{and} \quad h(\theta - \varphi) = \begin{pmatrix} \cos(\theta - \varphi) \\ \sin(\theta - \varphi) \\ 0 \\ 0 \end{pmatrix}. \tag{26}$$

Then, we have

$$g_\varphi \cdot h(\theta) = \begin{pmatrix} \cos(\varphi) & \sin(\varphi) & 0 & 0 \\ -\sin(\varphi) & \cos(\varphi) & 0 & 0 \\ 0 & 0 & 0 & 0 \\ 0 & 0 & 0 & 0 \end{pmatrix} h(\theta). \tag{27}$$

If instead we have that

$$\theta \in [0, \pi/2), \varphi \in [\theta, \theta + \pi/2), \tag{28}$$

such that

$$h(\theta) = \begin{pmatrix} \cos\theta \\ \sin\theta \\ 0 \\ 0 \end{pmatrix} \quad \text{and} \quad h(\theta - \varphi) = \begin{pmatrix} \cos(\theta - \varphi) \\ 0 \\ 0 \\ -\sin(\theta - \varphi) \end{pmatrix}, \tag{29}$$

then

$$g_\varphi \cdot h(\theta) = \begin{pmatrix} \cos(\varphi) & \sin(\varphi) & 0 & 0 \\ 0 & 0 & 0 & 0 \\ 0 & 0 & 0 & 0 \\ \sin(\varphi) & -\cos(\varphi) & 0 & 0 \end{pmatrix} h(\theta). \tag{30}$$

One could enumerate the matrices for each linear region, but we will not do so.

We note that this shows that the representation $h(\theta)$ is not in fact a linear representation in the group-theoretic sense, as the group action is not linear.

A.4 Alignment between two RSMs

Consider two instances of the four-neuron representation, with distinct gauge angles φ_1 and φ_2. Using the form of the RSM from (3), we can work out that the raw kernel alignment between their RSMs is

$$\begin{aligned} \mathrm{KA}(\varphi_1, \varphi_2) &= \frac{\mathrm{tr}(RSM_{\varphi_1} RSM_{\varphi_2})}{\sqrt{\mathrm{tr}(RSM_{\varphi_1} RSM_{\varphi_1})\,\mathrm{tr}(RSM_{\varphi_2} RSM_{\varphi_2})}} && (31) \\ &= \frac{1 + 2\rho(\varphi_1)\rho(\varphi_2)}{\sqrt{(1 + 2\rho(\varphi_1)^2)(1 + 2\rho(\varphi_2)^2)}} && (32) \end{aligned}$$

where $\rho(\varphi) = \sin\varphi\cos\varphi$ with φ taken modulo $\pi/2$. Defining the 4×4 centering matrix C by $C_{ij} = \delta_{ij} - 1/4$, the centered kernel alignment [12] is

$$\begin{aligned} \mathrm{CKA}(\varphi_1, \varphi_2) &= \frac{\mathrm{tr}(CRSM_{\varphi_1} CRSM_{\varphi_2})}{\sqrt{\mathrm{tr}(CRSM_{\varphi_1} CRSM_{\varphi_1})\,\mathrm{tr}(CRSM_{\varphi_2} CRSM_{\varphi_2})}} && (33) \\ &= \frac{3 - 2(\rho(\varphi_1) + \rho(\varphi_2)) + 4\rho(\varphi_1)\rho(\varphi_2)}{\sqrt{(3 - 4\rho(\varphi_1) + 4\rho(\varphi_1)^2)(3 - 4\rho(\varphi_2) + 4\rho(\varphi_2)^2)}}. && (34) \end{aligned}$$

Both of these expressions are gauge-dependent. Because of the properties of $\rho(\varphi)$, they are doubly-periodic, and both are minimized when $\rho(\varphi_1)$ and $\rho(\varphi_2)$ are maximally distinct. This occurs when one of φ_1 or φ_2 is zero modulo $\pi/2$, while the other is $\pi/4$ modulo $\pi/2$, which leads to either $\rho(\varphi_1)$ or $\rho(\varphi_2)$ vanishing while the other is equal to $1/2$. In such a case both the raw KA and the CKA are equal to $\sqrt{2/3} \approx 0.82$. We plot the resulting matrices of centered and non-centered kernel alignment in Figure S2.

B Deferred derivations related to general formalism

B.1 Gauge-invariance of decoding accuracy

Here, we show in detail how the gauge-invariance of the decoding error follows from the setup. As in the main text, Z is a latent space upon which the group G acts, equipped with a G-invariant probability measure μ_Z. An observation $x = x(z, \xi)$ is the result of applying an invertible function to $z \in Z$ and $\xi \in \Xi$, where Ξ is equipped with a probability measure μ_Ξ. We define $g \cdot x = x(g \cdot z, \xi)$ for any $x \in X$ using the fact that each element is uniquely associated to a pair (z, ξ). We assume X is equipped with a metric d_X that is G-invariant under this left action. The encoder h maps X to H, and we define $(g \cdot h)(x) = h(g^{-1} \cdot x) = h(x(g^{-1} \cdot z, \xi))$. In turn, the decoder f maps H to (a subset of) X. As we define the action of G on X, we can apply this definition to define an action of G on the decoding of a given representation, *i.e.*, we can define $(g \cdot f)(a) = g \cdot f(a)$ for $a \in H$. Note that this contrasts with the usual definition of a group acting on the inputs to a function.

As in the main text, define

$$\mathcal{E}_{avg}[h, f] = \int d_X(x(z, \xi), f(h(x(z, \xi))))\, d\mu_Z(z)\, d\mu_\Xi(\xi). \tag{35}$$

Our goal is to show that for any $g \in G$, we have

$$\mathcal{E}_{avg}[g \cdot h, g \cdot f] = \mathcal{E}_{avg}[h, f]. \tag{36}$$

For any $g \in G$, we have

$$\begin{aligned}
&\mathcal{E}_{avg}[g \cdot h, g \cdot f] \\
&= \int d_X(x(z, \xi), (g \cdot f)((g \cdot h)(x(z, \xi))))\, d\mu_Z(z)\, d\mu_\Xi(\xi) && (37) \\
&= \int d_X(x(z, \xi), (g \cdot f)(h(x(g^{-1} \cdot z, \xi))))\, d\mu_Z(z)\, d\mu_\Xi(\xi) \quad \text{(def. of } g \cdot h) && (38) \\
&= \int d_X(x(g^{-1} \cdot z, \xi), f(h(x(g^{-1} \cdot z, \xi))))\, d\mu_Z(z)\, d\mu_\Xi(\xi) \quad (G\text{-inv. of } d_X \text{ \& def. of } g^{-1} \cdot x) && (39) \\
&= \int d_X(x(z, \xi), f(h(x(z, \xi))))\, d\mu_Z(z)\, d\mu_\Xi(\xi) \quad (G\text{-invariance of } \mu_Z) && (40) \\
&= \mathcal{E}_{avg}[h, f] \quad \text{(def. of } \mathcal{E}_{avg}[h, f]). && (41)
\end{aligned}$$

This is the desired claim.

We note that the same logic applies to a case in which we replace the average over z with a supremum over z, or some mixture of averages and suprema over z and ξ. Concretely, if we define

$$\mathcal{E}_{sup}[h, f] = \sup_{z \in Z} \sup_{\xi \in \Xi} d_X(x(z, \xi), f(h(x(z, \xi)))), \tag{42}$$

we have

$$\begin{aligned}
\mathcal{E}_{sup}[g \cdot h, g \cdot f] &= \sup_{z \in Z} \sup_{\xi \in \Xi} d_X(x(z, \xi), (g \cdot f)((g \cdot h)(x(z, \xi)))) && (43) \\
&= \sup_{z \in Z} \sup_{\xi \in \Xi} d_X(x(g^{-1} \cdot z, \xi), f(h(x(g^{-1} \cdot z, \xi)))) && (44)
\end{aligned}$$

by an argument identical to the above. Now, taking the supremum over a function of $g^{-1} \cdot z$ with respect to z for some fixed g is no different than taking the supremum over z without the group transformation, so we have

$$\mathcal{E}_{sup}[g \cdot h, g \cdot f] = \mathcal{E}_{sup}[h, f]. \tag{45}$$

B.2 Conditions for gauge-invariance of the RSM

Here, we prove the claim of Section 3.3, which gives the conditions under which the RSM is gauge-invariant. Recall that our goal is to show that, for some fixed encoder h,

$$RSM_{g \cdot h}(x, x') = RSM_h(x, x') \tag{46}$$

for any two inputs $x, x' \in X$ and any group element $g \in G$ if and only if there exists an orthogonal matrix $O(g) \in \mathcal{O}(n)$, depending on g but not x, such that

$$(g \cdot h)(x) = O(g)h(x) \tag{47}$$

for all $x \in X$. The matrix $O(g)$ is not constrained outside the subspace $V = \text{span}\{h(x) : x \in X\}$ spanned by the representations. However, this non-uniqueness is relevant neither for the RSM nor for readout functionality.

It is immediately clear that it is sufficient for (47) to be satisfied for the RSM to be gauge-invariant, as under (47) we have

$$RSM_{g \cdot h}(x, x') = h(x)^\top O(g)^\top O(g)h(x') = h(x)^\top h(x') = RSM_h(x, x'). \tag{48}$$

What remains is to show that this condition is necessary. Fix a group element $g \in G$. Writing

$$\tilde{h}(x) = (g \cdot h)(x), \tag{49}$$

we assume that the RSM is gauge-invariant, so that

$$\tilde{h}(x)^\top \tilde{h}(x') = h(x)^\top h(x') \tag{50}$$

for all $x, x' \in X$. Let

$$V = \text{span}\{h(x) : x \in X\} \subseteq \mathbb{R}^n \tag{51}$$

be the linear subspace of $\mathbb{R}^n$ spanned by the representation. Because V is finite-dimensional, we may choose a finite set of $p \leq n$ inputs $\{x_1, \dots, x_p\}$ such that $\{h(x_1), \dots, h(x_p)\}$ is a basis for V. Consider the gauge-transformed representations $\{\tilde{h}(x_1), \dots, \tilde{h}(x_p)\}$. By assumption, they have the same Gram matrix as the basis $\{h(x_1), \dots, h(x_p)\}$. It is a classical fact (see Theorem 7.3.11 of Horn and Johnson [19]) that two real vector realizations of the same Gram matrix are related by an orthogonal transformation, *i.e.*, there must exist a matrix $O = O(g) \in \mathcal{O}(n)$ such that

$$\tilde{h}(x_i) = O(g)h(x_i), \quad i \in [p], \tag{52}$$

where we emphasize that the matrix is allowed to depend on g. What remains is to show that $Oh(x) = \tilde{h}(x)$ for all $x \in X$. Fixing an input $x \in X$, we therefore want to show that

$$\|\tilde{h}(x) - Oh(x)\|^2 = \tilde{h}(x)^\top \tilde{h}(x) - 2\tilde{h}(x)^\top Oh(x) + h(x)^\top O^\top Oh(x) \tag{53}$$

vanishes. By the assumption of RSM invariance, $\tilde{h}(x)^\top \tilde{h}(x) = h(x)^\top h(x)$, and by the fact that O is orthogonal, $h(x)^\top O^\top Oh(x) = h(x)^\top h(x)$. This leaves us with the cross term. Because $h(x) \in V$, we can expand it in the basis $\{h(x_1), \dots, h(x_p)\}$ as

$$h(x) = \sum_{i=1}^{p} a_i h(x_i) \tag{54}$$

for some set of coefficients $a_1, \dots, a_p$. Then, the cross term expands as

$$\tilde{h}(x)^\top Oh(x) = \sum_{i=1}^{p} a_i \tilde{h}(x)^\top Oh(x_i) \tag{55}$$

$$= \sum_{i=1}^{p} a_i \tilde{h}(x)^\top \tilde{h}(x_i) \qquad (\tilde{h}(x_i) = Oh(x_i)) \tag{56}$$

$$= \sum_{i=1}^{p} a_i h(x)^\top h(x_i) \qquad \text{(RSM invariance)} \tag{57}$$

$$= h(x)^\top h(x) \qquad \text{(basis expansion of } h(x)\text{)}. \tag{58}$$

Therefore, we have

$$\|\tilde{h}(x) - Oh(x)\|^2 = \tilde{h}(x)^\top \tilde{h}(x) - 2\tilde{h}(x)^\top Oh(x) + h(x)^\top O^\top Oh(x) \tag{59}$$

$$= h(x)^\top h(x) - 2h(x)^\top h(x) + h(x)^\top h(x) \tag{60}$$

$$= 0, \tag{61}$$

which proves that $\tilde{h}(x) = O(g)h(x)$ for the arbitrary input $x \in X$, and thus for all inputs. Importantly, the above argument shows that the matrix O is determined only on the span V of the representation, not on the orthogonal subspace $V^\perp$. We therefore conclude the desired claim.

In the analysis above, our starting assumption was that the full RSM was invariant for any pair of stimuli $x, x' \in X$. If we instead observed equality of the finite RSMs resulting from a set of trial stimuli, we could not guarantee that (47) holds globally. If the trial stimuli form a spanning set in the sense that they span V, then one could reconstruct the candidate matrix O, but showing that this extends to all un-tested stimuli requires stronger assumptions. In the proof above, this was possible thanks to the assumption of invariance of the RSM as a kernel function.

An alternative sufficient condition would be that we can write the full representation as an interpolant between the representations of the trial stimuli, *i.e.*, if there exist weights $\omega_i : X \to \mathbb{R}$ such that

$$h(x) = \sum_{i=1}^{p} \omega_i(x) h(x_i) \tag{62}$$

for any $x \in X$, where $\{x_1, \dots, x_p\}$ is the fixed set of trial stimuli on which we know the RSM is invariant. These weight functions must satisfy

$$\omega_i(x_j) = \delta_{ij} \tag{63}$$

for any probe stimulus, and crucially must be shared by the original and transformed representations, in the sense that

$$\tilde{h}(x) = \sum_{i=1}^{p} \omega_i(x) \tilde{h}(x_i) \tag{64}$$

for any $x \in X$. Then, supposing that the RSMs for the trial stimuli are invariant, we know that there is an O such that $\tilde{h}(x_i) = Oh(x_i)$ for all $i \in [p]$, and thus

$$\tilde{h}(x) = \sum_{i=1}^{p} \omega_i(x) \tilde{h}(x_i) = \sum_{i=1}^{p} \omega_i(x) O h(x_i) = O \sum_{i=1}^{p} \omega_i(x) h(x_i) = Oh(x) \tag{65}$$

for any $x \in X$.

B.3 Similarity matching between the stimulus and the representation

A natural question is whether objectives that explicitly aim to match the RSM to the stimulus similarity can fix a gauge. The simplest among such objective functions would be similarity matching [16, 33, 35], which would seek to minimize a population loss of the general form

$$\mathcal{C}[h] = \mathbb{E}_{z,z' \sim \mu_Z, \xi, \xi' \sim \mu_\Xi}[(k(z, z') - h(x)^\top h(x'))^2] \tag{66}$$

where $x = x(z, \xi)$ and $x' = x(z', \xi')$. Here, we assume that the latent space is equipped with a G-invariant similarity function k, *i.e.*, $k(\cdot, \cdot)$ is a symmetric function that satisfies

$$k(g \cdot z, g \cdot z') = k(z, z') \tag{67}$$

for any $g \in G$ and any $z, z' \in Z$. We claim that such a loss is gauge-invariant, in the sense that

$$\mathcal{C}[g \cdot h] = \mathcal{C}[h] \tag{68}$$

for any $g \in G$. The proof is straightforward:

$$\begin{aligned}
\mathcal{C}[g \cdot h] &= \mathbb{E}[(k(z, z') - (g \cdot h)(x(z, \xi))^\top (g \cdot h)(x(z', \xi')))^2] && (69)\\
&= \mathbb{E}[(k(z, z') - h(x(g^{-1} \cdot z, \xi))^\top h(x(g^{-1} \cdot z', \xi')))^2] && \text{(def. of } g \cdot h) \quad (70)\\
&= \mathbb{E}[(k(g \cdot z, g \cdot z') - h(x(z, \xi))^\top h(x(z', \xi')))^2] && (G\text{-inv. of } \mu_Z) \quad (71)\\
&= \mathcal{C}[h] && (G\text{-inv. of } k). \quad (72)
\end{aligned}$$

Now, in practice, one minimizes an empirical loss for a finite number of samples; this could in principle break the symmetry. However, as illustrated in Figure S3, even with a relatively modest number of examples the similarity matching loss can remain nearly gauge-independent.

C Detailed analysis of the toy orientation tuning model with many neurons

In the main text, we focused on the case where the toy orientation tuning model has only $n = 4$ neurons. This is the minimum number of neurons required to faithfully reconstruct the ring because (1) at least one neuron must respond to every stimulus, and (2) if only a single neuron responds to some set of stimuli, then responses may be ambiguous due to the symmetry in each neuron's response under reflection about the axis of its preferred stimulus. This, for instance, means that $n = 3$ neurons does not suffice, as with equidistributed receptive field centers there are thus regions around each neuron's receptive field center where only a single neuron is active. In this Appendix, we consider $n > 4$ neurons. We show that the RSM for the toy orientation tuning model has a simple form whenever the number of neurons is an integer multiple of four.

As in the main text, the activation of the i-th of n neurons is given by $h_i(x) = \mathrm{ReLU}(w_i^\top x)$, where $w_i = (\cos\theta_i, \sin\theta_i)^\top$ for $\theta_i = 2(i-1)\pi/n + \varphi$. The four test stimuli of interest are $s_1 = (1,0)^\top$, $s_2 = (0,1)^\top$, $s_3 = (-1,0)^\top$, and $s_4 = (0,-1)^\top$, and the RSM has elements $RSM_{ab} = \sum_{i=1}^n h_i(s_a)h_i(s_b)$ for $a, b \in [4]$.

C.1 Reconstruction

Before considering the RSM, we show that reconstruction is gauge-invariant for any even n. Let $n = 2m$. Then, for any φ, we can write the weight matrix as

$$W = \begin{pmatrix} \cos(\varphi) & \sin(\varphi) \\ \cos(\pi/m+\varphi) & \sin(\pi/m+\varphi) \\ \vdots & \\ \cos(\pi(m-1)/m+\varphi) & \sin(\pi(m-1)/m+\varphi) \\ \cos(\pi+\varphi) & \sin(\pi+\varphi) \\ \cos(\pi+\pi/m+\varphi) & \sin(\pi+\pi/m+\varphi) \\ \vdots & \\ \cos(\pi+\pi(m-1)/m+\varphi) & \sin(\pi+\pi(m-1)/m+\varphi) \end{pmatrix} = \sqrt{\frac{m}{2}} \begin{pmatrix} U \\ -U \end{pmatrix}, \tag{73}$$

where

$$U = \sqrt{\frac{2}{m}} \begin{pmatrix} \cos(\varphi) & \sin(\varphi) \\ \cos(\pi/m+\varphi) & \sin(\pi/m+\varphi) \\ \vdots & \\ \cos(\pi(m-1)/m+\varphi) & \sin(\pi(m-1)/m+\varphi) \end{pmatrix} \in \mathbb{R}^{m\times 2}. \tag{74}$$

The matrix U has the important property that it is semi-orthogonal:

$$U^\top U = I_2, \tag{75}$$

as can be verified by computing each of its elements:

$$(U^\top U)_{11} = \frac{2}{m}\sum_{k=0}^{m-1} \cos(\pi k/m+\varphi)^2 = 1 \tag{76}$$

$$(U^\top U)_{12} = (U^\top U)_{21} = \frac{2}{m}\sum_{k=0}^{m-1} \cos(\pi k/m+\varphi)\sin(\pi k/m+\varphi) = 0 \tag{77}$$

$$(U^\top U)_{22} = \frac{2}{m}\sum_{k=0}^{m-1} \sin(\pi k/m+\varphi)^2 = 1. \tag{78}$$

Using the general result we prove in Appendix D.1 for reconstructions of the $(d-1)$-sphere $\mathbb{S}^{d-1}$ in the special case $d = 2$, we therefore conclude that perfect decoding for any gauge angle is possible by using $\frac{2}{m}W^\top$ as the decoding weights, where the normalization factor does not matter because the ReLU is positive-homogeneous. In other words, for any gauge angle φ we have

$$\frac{2}{m}W^\top \mathrm{ReLU}(Wx) = x \tag{79}$$

for all $x \in \mathbb{S}^1$.

C.2 Evaluation of the similarity matrix elements

By symmetry, all diagonal elements of the RSM are equal to:

$$RSM_{diag} = \sum_{k=0}^{n-1} \mathrm{ReLU}\left(\cos\left(\frac{2\pi k}{n} + \varphi\right)\right)^2, \tag{80}$$

and non-zero off-diagonal elements are all equal to:

$$\rho = \sum_{k=0}^{n-1} \mathrm{ReLU}\left(\cos\left(\frac{2\pi k}{n} + \varphi\right)\right) \mathrm{ReLU}\left(\sin\left(\frac{2\pi k}{n} + \varphi\right)\right). \tag{81}$$

We can see that increasing φ by integer multiples of $2\pi/n$ is equivalent to shifting the index k, which has the effect of circularly permuting neuron labels. As the RSM is invariant under permutation of the neuron labels, it must therefore be periodic in φ, with period $2\pi/n$. Therefore, it suffices to consider $\varphi \in [0, 2\pi/n)$. We therefore put $\varphi = 2\pi\delta/n$ for $\delta \in [0, 1)$.

First, consider the off-diagonal elements. As $\cos(\theta)$ and $\sin(\theta)$ are simultaneously positive only for $\theta \in [0, \pi/2)$, this is equivalent to

$$\rho = \sum_{k=0}^{n-1} \cos\left(\frac{2\pi(k+\delta)}{n}\right) \sin\left(\frac{2\pi(k+\delta)}{n}\right) \mathbf{1}_{0 \le k+\delta < n/4} \tag{82}$$

Supposing that $n = 4q$ for an integer $q \ge 1$, we see that $0 \le k + \delta < n/4$ only if $k \le q - 1$, for any $\delta \in [0, 1)$. Therefore,

$$\rho = \sum_{k=0}^{q-1} \cos\left(\frac{\pi(k+\delta)}{2q}\right) \sin\left(\frac{\pi(k+\delta)}{2q}\right) \tag{83}$$

This is at last a sum that can be evaluated, giving

$$\rho = \frac{1}{2}\cot\left(\frac{\pi}{2q}\right)\cos\left(\frac{\pi\delta}{q}\right) + \frac{1}{2}\sin\left(\frac{\pi\delta}{q}\right) \tag{84}$$

or, in terms of n and φ,

$$\rho = \frac{1}{2}\cot\left(\frac{2\pi}{n}\right)\cos(2\varphi) + \frac{1}{2}\sin(2\varphi). \tag{85}$$

As $\cos(\theta)$ is positive if $\theta \in [0, \pi/2) \cup (3\pi/2, 2\pi)$, we can similarly evaluate the diagonal elements as

$$\begin{aligned} &\sum_{k=0}^{n-1} \cos\left(\frac{2\pi(k+\delta)}{n}\right)^2 [\mathbf{1}_{0 \le k+\delta < q} + \mathbf{1}_{3q < k+\delta < 4q}] && (86) \\ &= \sum_{k=0}^{q-1} \cos\left(\frac{\pi(k+\delta)}{2q}\right)^2 + \sum_{k=3q}^{4q-1} \cos\left(\frac{\pi(k+\delta)}{2q}\right)^2 && (87) \\ &= q. && (88) \end{aligned}$$

Thus, letting

$$\rho_n(\varphi) = \frac{1}{2}\cot\left(\frac{2\pi}{n}\right)\cos(2\varphi) + \frac{1}{2}\sin(2\varphi), \tag{89}$$

we find that for any n a multiple of four we have

$$RSM = \begin{pmatrix} \frac{n}{4} & \rho_n(\varphi) & 0 & \rho_n(\varphi) \\ \rho_n(\varphi) & \frac{n}{4} & \rho_n(\varphi) & 0 \\ 0 & \rho_n(\varphi) & \frac{n}{4} & \rho_n(\varphi) \\ \rho_n(\varphi) & 0 & \rho_n(\varphi) & \frac{n}{4} \end{pmatrix} \tag{90}$$

for any $\varphi \in [0, 2\pi/n)$. For larger φ, we can apply this result using $\varphi \mod 2\pi/n$.

We can evaluate by a similar argument the population-averaged firing rate for each of the test stimuli:

$$\bar{r} = \frac{1}{n}\sum_{k=0}^{n-1}\cos\left(\frac{2\pi(k+\delta)}{n}\right)[\mathbf{1}_{0\leq k+\delta<q} + \mathbf{1}_{3q<k+\delta<4q}] \tag{91}$$

$$= \frac{1}{n}\sum_{k=0}^{q-1}\cos\left(\frac{\pi(k+\delta)}{2q}\right) + \frac{1}{n}\sum_{k=3q}^{4q-1}\cos\left(\frac{\pi(k+\delta)}{2q}\right) \tag{92}$$

$$= \frac{1}{n}\csc\left(\frac{\pi}{4q}\right)\cos\left(\frac{\pi - 2\pi\delta}{4q}\right) \tag{93}$$

$$= \frac{1}{n}\csc\left(\frac{\pi}{n}\right)\cos\left(\frac{\pi}{n} - \varphi\right), \tag{94}$$

where again in the last line we consider $\varphi \in [0, 2\pi/n)$.

C.3 Large-n limit

We now want to study what happens when we take the number of neurons to be large. To do so, it is convenient to use the parameterization of the mean firing rate and RSM in terms of q and δ, as the period of these objects in δ is defined to be independent of n. First, the mean firing rate expands as

$$\bar{r} = \frac{1}{4q}\csc\left(\frac{\pi}{4q}\right)\cos\left(\frac{\pi - 2\pi\delta}{4q}\right) \tag{95}$$

$$= \frac{1}{\pi} - \frac{\pi(1 - 6\delta + 6\delta^2)}{48q^2} + \mathcal{O}\left(\frac{1}{q^4}\right). \tag{96}$$

The normalized non-zero off-diagonal elements of the RSM are given by

$$\frac{1}{2q}\cot\left(\frac{\pi}{2q}\right)\cos\left(\frac{\pi\delta}{q}\right) + \frac{1}{2q}\sin\left(\frac{\pi\delta}{q}\right) = \frac{1}{\pi} - \frac{\pi(1 - 6\delta + 6\delta^2)}{12q^2} + \mathcal{O}\left(\frac{1}{q^4}\right). \tag{97}$$

This shows that the mean firing rate and RSM are gauge-invariant only asymptotically, in the limit $n \to \infty$.

C.4 Normalized variability

As in the main text, we consider the normalized variability

$$\Delta_n = \frac{\max_\varphi RSM_{off} - \min_\varphi RSM_{off}}{RSM_{diag}}. \tag{98}$$

Using the expression for RSM_{off} from above, we see that it is maximized at $\delta = 1/2$, where it takes value

$$\frac{1}{2}\cot\left(\frac{\pi}{2q}\right)\cos\left(\frac{\pi\delta}{q}\right) + \frac{1}{2}\sin\left(\frac{\pi\delta}{q}\right)\bigg|_{\delta=1/2} = \frac{1}{2}\csc\left(\frac{\pi}{2q}\right) \tag{99}$$

and minimized at $\delta = 0$, where it takes value

$$\frac{1}{2}\cot\left(\frac{\pi}{2q}\right)\cos\left(\frac{\pi\delta}{q}\right) + \frac{1}{2}\sin\left(\frac{\pi\delta}{q}\right)\bigg|_{\delta=0} = \frac{1}{2}\cot\left(\frac{\pi}{2q}\right). \tag{100}$$

Using the identity $\csc(\theta) - \cot(\theta) = \tan(\theta/2)$, we thus have that

$$\Delta_{4q} = \frac{1}{2q}\tan\left(\frac{\pi}{4q}\right), \tag{101}$$

or

$$\Delta_n = \frac{2}{n}\tan\left(\frac{\pi}{n}\right) \tag{102}$$

in terms of n. As $\tan(\theta) = \theta + \mathcal{O}(\theta^3)$ as $\theta \downarrow 0$, we thus find that

$$\Delta_{4q} = \frac{\pi}{8q^2} + \mathcal{O}\left(\frac{1}{q^4}\right) \tag{103}$$

as $q \to \infty$, which is of course equivalent to

$$\Delta_n = \frac{2\pi}{n^2} + \mathcal{O}\left(\frac{1}{n^4}\right). \tag{104}$$

C.5 Penalizing the L_1 norm of activations does not fix a gauge

In the main text, we showed that one way of promoting RSM variability with a large number of neurons is the presence of energetic cost (such as the L1-penalty) on the activations. Here, we show that when data is uniformly distributed on the manifold, L1-penalty does not fix a gauge. Observe that the response of the i-th neuron to a stimulus $x = (\cos\psi, \sin\psi)^\top$ is simply

$$h_i(x) = \mathrm{ReLU}\left(\cos\left(\frac{2\pi(i-1)}{n} + \varphi - \psi\right)\right), \quad i \in [n]. \tag{105}$$

Then, the L_p norm of the hidden layer activations, assuming a uniform angular distribution, is

$$\|h\|_p = \left(\int_0^{2\pi} \frac{d\psi}{2\pi} \bar{r}_{n,p}(\varphi - \psi)\right)^{1/p}, \tag{106}$$

where we let

$$\bar{r}_{n,p}(\varphi - \psi) = \sum_{k=0}^{n-1} \mathrm{ReLU}\left(\cos\left(\frac{2\pi k}{n} + \varphi - \psi\right)\right)^p. \tag{107}$$

Though we have assumed that everything is normalized, we remark that if we allowed radial variation in the stimulus, we could ignore it in this analysis. This is because the positive-homogeneity of the ReLU means that adding a variable radius will simply multiply the formula above by a constant independent of both n and φ.

By an argument identical to our previous analysis, $\bar{r}_{n,p}(\varphi - \psi)$ must be $2\pi/n$-periodic in its argument. Therefore, by averaging over $\psi \in [0, 2\pi)$ we are averaging over n periods, which in turn means that $\|h\|_p$ must be independent of the gauge angle φ. Thus, penalizing the norm of the activations cannot fix a gauge.

How do deviations of the stimulus distribution from a uniform distribution on the full circle affect this result? In particular, what happens if we consider a discrete set of equally-spaced points on the circle, in which case the rotation symmetry is replaced by a cyclic group. Recalling our results from before, we have for n an integer multiple of four that

$$\bar{r}_{n,1}(\varphi - \psi) = \csc\left(\frac{\pi}{n}\right)\cos\left(\frac{\pi}{n} - (\varphi - \psi)\right), \tag{108}$$

where we should plug in the residue of $\varphi - \psi$ modulo $2\pi/n$, while

$$\bar{r}_{n,2}(\varphi - \psi) = \frac{n}{4}. \tag{109}$$

This means that for n a multiple of 4 penalizing the L_2 norm of activations cannot fix a gauge for the trivial reason that it is stimulus-independent. In contrast, the L_1 norm is in principle sensitive to fluctuations in the distribution of stimuli. To see whether this can fix a gauge in the case where the stimuli are discrete and equidistributed around the circle, first suppose that $n = 4$. Then,

$$\bar{r}_{4,1}(\phi - \psi) = \cos(\varphi - \psi) + \sin(\varphi - \psi) \tag{110}$$

must be evaluated using the residue of $\varphi - \psi$ modulo $\pi/2$. One can then, for instance, evaluate the total L_1 norm in the case where there are four stimuli equidistributed about the circle, which leads to

$$\|h\|_1 = \frac{1}{4}\sum_{k=0}^{3} \bar{r}_{4,1}(\varphi - \pi k/2) = \cos\varphi + \sin\varphi \tag{111}$$

for φ taken modulo $\pi/2$. This is clearly not gauge-invariant. However, it does not prefer a unique φ, only some discrete set. This generalizes to larger numbers of stimuli, where including a penalty leads to a quantized set of preferred gauge angles φ.

C.6 The RSM for arbitrary angular differences

We can extend the analysis above to cover arbitrary angular displacements. To cover the circle, we consider s_1 and

$$s_\alpha = [\cos(\alpha), \sin(\alpha)]^\top \tag{112}$$

for $-\pi \leq \alpha \leq \pi$. With n neurons, the RSM between the representations of the vectors s_1 and s_α is

$$RSM_{s_1,s_\alpha} = \sum_{k=0}^{n-1} \operatorname{ReLU}\left(\cos\left(\frac{2\pi k}{n} + \varphi\right)\right) \operatorname{ReLU}\left(\cos\left(\frac{2\pi k}{n} + \varphi - \alpha\right)\right). \tag{113}$$

By a rationale identical to our previous analysis, this is $2\pi/n$-periodic in the gauge angle φ, so it suffices to consider $\varphi \in [0, 2\pi/n)$. This holds for any α.

It is useful to observe that, in the $n \to \infty$ limit where the dependence on φ drops out, we have

$$\lim_{n\to\infty} \frac{1}{n} RSM_{s_1,s_\alpha} = \frac{1}{2\pi} \int_0^{2\pi} \operatorname{ReLU}(\cos(\theta)) \operatorname{ReLU}(\cos(\theta - \alpha))\, d\theta. \tag{114}$$

This is clearly a 2π-periodic function of α, and moreover is even in α as we are integrating over a full period. Note that this argument does not go through in the discrete case, as there is gauge-dependence when n is finite, and there negating α yields the same answer up to a shift of gauge $\varphi \mapsto \varphi - \alpha$. Returning to the continuum limit, it is then clear that it suffices to consider $\alpha \in [0, \pi]$. Then, we compute

$$\begin{aligned}
\lim_{n\to\infty} \frac{1}{n} RSM_{s_1,s_\alpha} &= \frac{1}{2\pi} \int_0^{\pi/2} \cos(\theta) \operatorname{ReLU}(\cos(\theta - \alpha))\, d\theta \\
&\quad + \frac{1}{2\pi} \int_{3\pi/2}^{2\pi} \cos(\theta) \operatorname{ReLU}(\cos(\theta - \alpha))\, d\theta && (115) \\
&= \frac{1}{2\pi} \int_{\max(0,\alpha-\pi/2)}^{\pi/2} \cos(\theta) \cos(\theta - \alpha)\, d\theta \\
&\quad + \frac{1}{2\pi} \int_{\min(2\pi,3\pi/2+\alpha)}^{2\pi} \cos(\theta) \cos(\theta - \alpha)\, d\theta && (116) \\
&= \frac{2\theta\cos(\alpha) + \sin(2\theta - \alpha)}{8\pi} \Bigg|_{\max(0,\alpha-\pi/2)}^{\pi/2} \\
&\quad + \frac{2\theta\cos(\alpha) + \sin(2\theta - \alpha)}{8\pi} \Bigg|_{\theta=\min(2\pi,3\pi/2+\alpha)}^{2\pi} && (117) \\
&= \frac{(\pi - \alpha)\cos(\alpha) + \sin(\alpha)}{4\pi}. && (118)
\end{aligned}$$

Using the symmetry and periodicity properties of the function, this determines its value for any α. Up to normalization, this is the familiar arc-cosine kernel from Cho and Saul [46], as we would expect.

We now return to the discrete computation, which is a straightforward but slightly tedious exercise in trigonometric sums. As before, we proceed by letting $n = 4q$ for some positive integer q, and by letting $\varphi = 2\pi\delta/n = \pi\delta/(2q)$ for some $\delta \in [0, 1)$. This leads to

$$RSM_{s_1,s_\alpha} = \sum_{k=0}^{4q-1} \operatorname{ReLU}\left(\cos\left(\frac{\pi(k+\delta)}{2q}\right)\right) \operatorname{ReLU}\left(\cos\left(\frac{\pi(k+\delta)}{2q} - \alpha\right)\right). \tag{119}$$

As $\cos(\theta)$ is positive if $\theta \in [0, \pi/2) \cup (3\pi/2, 2\pi)$, we can expand the summation based on when the first rectified cosine is non-zero as

$$RSM_{s_1,s_\alpha} = \sum_{k\in\{0,\dots,q-1\}\cup\{3q,\dots,4q-1\}} \cos\left(\frac{\pi(k+\delta)}{2q}\right) \operatorname{ReLU}\left(\cos\left(\frac{\pi(k+\delta)}{2q} - \alpha\right)\right). \tag{120}$$

We now make a further change of variables, and write

$$\varphi - \alpha = \frac{\pi}{2q}(m + \varepsilon) \tag{121}$$

where m is an integer and $\varepsilon \in [0, 1)$, in terms of which we have

$$RSM_{s_1,s_\alpha} = \sum_{k\in\{0,\dots,q-1\}\cup\{3q,\dots,4q-1\}} \cos\left(\frac{\pi(k+\delta)}{2q}\right) \mathrm{ReLU}\left(\cos\left(\frac{\pi(k+m+\varepsilon)}{2q}\right)\right). \tag{122}$$

As this function is $4q$-periodic in m, it is sufficient to consider the range $-2q \le m \le 2q$.

Consider the first range in the summation, with $k \in \{0, \dots, q-1\}$. Then, the second rectified cosine is simultaneously positive if k is such that

$$-\frac{\pi}{2} \le \frac{\pi}{2q}(k + m + \varepsilon) < \frac{\pi}{2}, \tag{123}$$

where the upper limit is open because $\cos(\pi/2) = 0$. Multiplying through by $2q/\pi$ and using the fact that $k + m$ and q are integers, this means we should have

$$-q \le k + m \le q - 1. \tag{124}$$

Therefore, the first interval of summation is truncated from $\{0, \dots, q-1\}$ to

$$I_1 = \{\max(0, -m-q), \dots, \min(q-1, q-m-1)\}, \tag{125}$$

which is an empty set if $m > q$.

Now consider the second range in the summation, with $k \in \{3q, \dots, 4q-1\}$. Then, the second rectified cosine is simultaneously positive if

$$\frac{3\pi}{2} \le \frac{\pi}{2q}(k + m + \varepsilon) < \frac{5\pi}{2}; \tag{126}$$

again the upper limit is open because $\cos(5\pi/2) = 0$. This simplifies to

$$3q \le k + m \le 5q - 1, \tag{127}$$

meaning that the second interval of summation is truncated from $\{3q, \dots, 4q-1\}$ to

$$I_2 = \{\max(3q, 3q-m), \dots, \min(4q-1, 5q-m-1)\}, \tag{128}$$

which is empty if $m < -q$.

We therefore at last have the summation

$$RSM_{s_1,s_\alpha} = \sum_{k\in I_1\cup I_2} \cos\left(\frac{\pi(k+\delta)}{2q}\right) \cos\left(\frac{\pi(k+m+\epsilon)}{2q}\right), \tag{129}$$

which can be evaluated and simplified using Mathematica, yielding

$$\begin{aligned} RSM_{s_1,s_\alpha} = {} & \frac{1}{4}\operatorname{sign}(m)\csc\left(\frac{\pi}{2q}\right)\left[\sin\left(\frac{\pi(m+1-\delta-\varepsilon)}{2q}\right) + \sin\left(\frac{\pi(m-1+\delta+\varepsilon)}{2q}\right)\right] \\ & + \frac{2q-|m|}{2}\cos\left(\frac{\pi(m-\delta+\varepsilon)}{2q}\right), \end{aligned} \tag{130}$$

using the convention $\operatorname{sign}(0) = 1$.

This result is slightly unwieldy, but we can verify that it satisfies several important sanity checks. First, we examine its behavior at large q, where we can compare to the asymptotic formula we found before. At large q, both δ and ε can be neglected, and we have

$$m \sim -\frac{2q}{\pi}\alpha. \tag{131}$$

Then, a brief computation shows that

$$\lim_{q\to\infty} \frac{1}{4q} RSM_{s_1,s_\alpha} = \frac{(\pi - |\alpha|)\cos(\alpha) + \sin(|\alpha|)}{4\pi}, \tag{132}$$

which agrees with the result we found before.

Second, if we set $\alpha = \pi/2$ by substituting in $m = -q$ and $\varepsilon = \delta$, we should recover the result we found before for RSM_{s_1,s_2}. In this case, the expression above simplifies to

$$\frac{1}{2}\csc\left(\frac{\pi}{2q}\right)\cos\left(\frac{\pi}{2q} - \frac{\pi\delta}{q}\right) = \frac{1}{2}\cot\left(\frac{\pi}{2q}\right)\cos\left(\frac{\pi\delta}{q}\right) + \frac{1}{2}\sin\left(\frac{\pi\delta}{q}\right), \tag{133}$$

which agrees with what we found before.

A third check comes from substituting in $\alpha = \pi$ by taking $m = -2q$ and $\varepsilon = \delta$, which corresponds to RSM_{s_1,s_3}. One can see that the expression we found above then vanishes as we would expect.

A fourth check comes from considering the four-neuron case $q = 1$, for which we computed RSM_{s_1,s_α} independently for $-\pi/2+\varphi < \alpha < \varphi$ above (for convenience we assume both inequalities are strict). For $q = 1$, we have $\varphi \in [0, \pi/2)$ and $\delta = 2\varphi/\pi$. Then, $m = \left\lfloor \frac{2}{\pi}(\varphi - \alpha) \right\rfloor = 0$ for all α of interest, and thus $\varepsilon = \frac{2}{\pi}(\varphi - \alpha)$. With all of this, the formula we computed simplifies to $RSM_{s_1,s_\alpha} = \cos(\alpha)$, matching what we found before.

A fifth check comes from setting $\alpha = 0$ by taking $m = 0$ and $\varepsilon = \delta$, which should recover $RSM_{s_1,s_1} = q$. It is easy to check that the formula we found agrees with this result.

With these checks out of the way, we now turn to the problem that the variables m and ε, while convenient for computation, are not very useful if we want to apply the end result. To do so, we need to substitute in

$$m = \left\lfloor \frac{2q}{\pi}(\varphi - \alpha) \right\rfloor = \left\lfloor \delta - \frac{2q}{\pi}\alpha \right\rfloor \tag{134}$$

and

$$\varepsilon = \frac{2q}{\pi}(\varphi - \alpha) - m = \delta - \frac{2q}{\pi}\alpha - \left\lfloor \delta - \frac{2q}{\pi}\alpha \right\rfloor. \tag{135}$$

This does not lead to substantial simplification of the formula, but allows it to be evaluated numerically and compared to experiments.

One important feature of this result is that RSM_{s_1,s_α} is in general an even function of α only in the limit $n \to \infty$. This property also holds in the special case $\varphi = 0$. The symmetry—or lack thereof—at finite n is clear geometrically. This is, importantly, not evident from our earlier results, which only considered α as an integer multiple of $\pi/2$. We can show that this extends to the case where α is any integer multiple of $\pi/2q$, *i.e.*, that there exists $r \in \{-2q, \ldots, 2q\}$ such that $\alpha = \pi r/2q$. Then,

$$m = \lfloor \delta - r \rfloor = \lfloor \delta \rfloor - r = -r \tag{136}$$

and

$$\varepsilon = \delta - r - m = \delta \tag{137}$$

With this, we can simplify the expression for the RSM to

$$RSM_{s_1,s_\alpha}\Big|_{\alpha=\frac{\pi r}{2q}} = \frac{1}{2}\csc\left(\frac{\pi}{2q}\right)\sin\left(\frac{\pi|r|}{2q}\right)\cos\left(\frac{\pi}{2q} - \frac{\pi\delta}{q}\right) + \frac{2q-|r|}{2}\cos\left(\frac{\pi r}{2q}\right), \tag{138}$$

which is an even function of r.

For values of α not satisfying this condition, the RSM is not in general even unless $\varphi = 0$. Algebraically, we can prove the symmetry with $\varphi = 0$ in a few steps. With $\varphi = 0$, we have

$$m = \left\lfloor -\frac{2q}{\pi}\alpha \right\rfloor \tag{139}$$

and

$$\varepsilon = -\frac{2q}{\pi}\alpha - m \tag{140}$$

so

$$\frac{\pi}{2q}(m + \varepsilon) = -\alpha \tag{141}$$

while

$$\frac{\pi}{2q}(m-\varepsilon)=\frac{\pi m}{q}+\alpha. \tag{142}$$

Thus,

$$\begin{aligned}RSM_{s_1,s_\alpha}\Big|_{\varphi=0} &= \frac{1}{4}\operatorname{sign}(m)\csc\left(\frac{\pi}{2q}\right)\left[\sin\left(\frac{\pi}{2q}+\frac{\pi m}{q}+\alpha\right)-\sin\left(\frac{\pi}{2q}+\alpha\right)\right]\\ &\quad+\frac{2q-|m|}{2}\cos(\alpha).\end{aligned} \tag{143}$$

Suppose that $\alpha>0$. Then,

$$m=-\left\lceil\frac{2q}{\pi}\alpha\right\rceil<0, \tag{144}$$

which leads to

$$\begin{aligned}RSM_{s_1,s_\alpha}\Big|_{\varphi=0} &= -\frac{1}{4}\csc\left(\frac{\pi}{2q}\right)\left[\sin\left(\frac{\pi}{2q}-\frac{\pi}{q}\left\lceil\frac{2q}{\pi}\alpha\right\rceil+\alpha\right)-\sin\left(\frac{\pi}{2q}+\alpha\right)\right]\\ &\quad+\left(q-\frac{1}{2}\left\lceil\frac{2q}{\pi}\alpha\right\rceil\right)\cos(\alpha).\end{aligned} \tag{145}$$

Now suppose that $\alpha<0$, so that

$$m=\left\lfloor\frac{2q}{\pi}|\alpha|\right\rfloor, \tag{146}$$

and thus

$$\begin{aligned}RSM_{s_1,s_\alpha}\Big|_{\varphi=0} &= \frac{1}{4}\csc\left(\frac{\pi}{2q}\right)\left[\sin\left(\frac{\pi}{2q}+\frac{\pi}{q}\left\lfloor\frac{2q}{\pi}|\alpha|\right\rfloor-|\alpha|\right)-\sin\left(\frac{\pi}{2q}-|\alpha|\right)\right]\\ &\quad+\left(q-\frac{1}{2}\left\lfloor\frac{2q}{\pi}|\alpha|\right\rfloor\right)\cos(|\alpha|).\end{aligned} \tag{147}$$

Then, for $0<\alpha\le\pi$, we have

$$\begin{aligned}&\left(RSM_{s_1,s_{-\alpha}}-RSM_{s_1,s_\alpha}\right)\Big|_{\varphi=0}\\ &=\frac{1}{4}\csc\left(\frac{\pi}{2q}\right)\left[\sin\left(\frac{\pi}{2q}+\frac{\pi}{q}\left\lfloor\frac{2q}{\pi}\alpha\right\rfloor-\alpha\right)-\sin\left(\frac{\pi}{2q}-\alpha\right)\right.\\ &\qquad\left.+\sin\left(\frac{\pi}{2q}-\frac{\pi}{q}\left\lceil\frac{2q}{\pi}\alpha\right\rceil+\alpha\right)-\sin\left(\frac{\pi}{2q}+\alpha\right)\right]\\ &\quad-\frac{1}{2}\left(\left\lfloor\frac{2q}{\pi}\alpha\right\rfloor-\left\lceil\frac{2q}{\pi}\alpha\right\rceil\right)\cos(\alpha)\end{aligned} \tag{148}$$

First consider the special case in which α is precisely an integer multiple of $\pi/2q$, *i.e.*, that there is an integer $r\in\{0,1,\ldots,2q\}$ such that

$$\alpha=\frac{\pi r}{2q}. \tag{149}$$

Then,

$$\left\lfloor\frac{2q}{\pi}\alpha\right\rfloor=\left\lceil\frac{2q}{\pi}\alpha\right\rceil=r, \tag{150}$$

and we have the simplification

$$\left(RSM_{s_1,s_{-\alpha}}-RSM_{s_1,s_\alpha}\right)\Big|_{\varphi=0}=\frac{1}{2}\left[\cos\left(\frac{\pi r}{q}-\frac{\pi r}{2q}\right)-\cos\left(\frac{\pi r}{2q}\right)\right] \tag{151}$$

$$=0. \tag{152}$$

If α is not an integer multiple of $\pi/2q$, then there is an integer $r \in \{0, \ldots, 2q-1\}$ such that

$$\left\lceil \frac{2q}{\pi}\alpha \right\rceil = \left\lfloor \frac{2q}{\pi}\alpha \right\rfloor + 1 \equiv r + 1. \tag{153}$$

Then, we have

$$\left(RSM_{s_1,s_{-\alpha}} - RSM_{s_1,s_\alpha}\right)\Big|_{\varphi=0} = -\frac{1}{2}\cos(\alpha) + \frac{1}{2}\cos(\alpha) = 0. \tag{154}$$

Therefore, $RSM_{s_1,s_\alpha}|_{\varphi=0}$ is an even function of α.

C.7 Amplitude variability

We can also add other sources of RSM variability to the toy model. For example, we can let the amplitudes of the receptive fields vary, as would result from weights of fluctuating norm. Suppose in general that

$$h_i(x) = a_i \operatorname{ReLU}(w_i^\top x) \tag{155}$$

for some non-negative amplitude a_i, such that

$$RSM_{s_1,s_\alpha} = \sum_{k=0}^{n-1} a_k^2 \operatorname{ReLU}\left(\cos\left(\frac{2\pi k}{n} + \varphi\right)\right) \operatorname{ReLU}\left(\cos\left(\frac{2\pi k}{n} + \varphi - \alpha\right)\right). \tag{156}$$

When all amplitudes are identical, then the RSM is a $2\pi/n$-periodic function of the gauge angle φ. This is because with equal amplitudes shifting the gauge angle by an integer multiple of $2\pi/n$ corresponds to circularly shifting the neurons, which does not change the RSM. Now, this circular shift also changes the amplitudes, and therefore can change the RSM.

This can be easily seen by examining RSM_{s_1,s_2} with $n = 4$ neurons, for which we have

$$RSM_{s_1,s_2} = |\sin(\varphi)\cos(\varphi)| \times \begin{cases} a_1^2, & 0 \le \varphi < \pi/2 \\ a_4^2, & \pi/2 \le \varphi < \pi \\ a_3^2, & \pi \le \varphi < 3\pi/2 \\ a_2^2, & 3\pi/2 \le \varphi < 2\pi. \end{cases} \tag{157}$$

In general, we can proceed by writing

$$\varphi = \frac{2\pi}{n}(m + \delta) \tag{158}$$

in terms of $m \in \{0, \ldots, n-1\}$ and $\delta \in [0, 1)$, and interpreting the indices of the amplitudes modulo n. Then, further assuming that $n = 4q$, we have

$$\begin{aligned} RSM_{s_1,s_2} &= \sum_{k=0}^{n-1} a_k^2 \operatorname{ReLU}\left(\cos\left(\frac{2\pi k}{n} + \varphi\right)\right) \operatorname{ReLU}\left(\sin\left(\frac{2\pi k}{n} + \varphi\right)\right) && (159) \\ &= \sum_{k=0}^{n-1} a_{k-m}^2 \operatorname{ReLU}\left(\cos\left(\frac{\pi(k+\delta)}{2q}\right)\right) \operatorname{ReLU}\left(\sin\left(\frac{\pi(k+\delta)}{2q}\right)\right) && (160) \\ &= \sum_{k=0}^{q-1} a_{k-m}^2 \cos\left(\frac{\pi(k+\delta)}{2q}\right) \sin\left(\frac{\pi(k+\delta)}{2q}\right) && (161) \end{aligned}$$

and

$$\begin{aligned} RSM_{s_1,s_1} &= \sum_{k=0}^{n-1} a_k^2 \operatorname{ReLU}\left(\cos\left(\frac{2\pi k}{n} + \varphi\right)\right)^2 && (162) \\ &= \sum_{k=0}^{n-1} a_{k-m}^2 \operatorname{ReLU}\left(\cos\left(\frac{2\pi(k+\delta)}{n}\right)\right)^2 && (163) \\ &= \sum_{k=0}^{q-1} a_{k-m}^2 \cos\left(\frac{\pi(k+\delta)}{2q}\right)^2 + \sum_{k=3q}^{4q-1} a_{k-m}^2 \cos\left(\frac{\pi(k+\delta)}{2q}\right)^2. && (164) \end{aligned}$$

In general, these expressions do not have simple closed forms. However, we can see that RSM_{s_1,s_2} is now not an even function of δ with its maximum at $\delta = 1/2$, as was true with equal amplitudes.

D Reflection-symmetric tilings of the sphere in higher dimensions

Performing a similarly-detailed analysis of the RSMs for tiling representations of higher-dimensional manifolds is challenging. Even for the ordinary sphere $\mathbb{S}^2$, explicitly writing down a tiling solution for an arbitrary number of neurons is challenging—this corresponds to a variant of the classic Thompson problem in potential theory, to which the general solution remains unknown [21].

Though we cannot write down the RSM explicitly, we can characterize its overall structure for solutions with an even number of neurons tiling the sphere $\mathbb{S}^{d-1}$ in d dimensions that obey a reflection-symmetry condition on the weights: for each neuron, there is another neuron with its receptive field oriented exactly opposite to the first. Let the number of neurons be $n = 2N$, and assume that the stimulus-by-neuron weight matrix $W \in \mathbb{R}^{d\times 2N}$ has the form

$$W = (U^\top, -U^\top) \tag{165}$$

for some matrix $U \in \mathbb{R}^{N\times d}$.[4] Generalizing our study of the $d = 2$ case, we take our probe stimuli to be the standard basis vectors and their negations, *i.e.*, we have a stimulus matrix[5]

$$S = (I_d, -I_d) \in \mathbb{R}^{d\times 2d}, \tag{166}$$

whose representation is

$$H = \mathrm{ReLU}(W^\top S) = \begin{pmatrix} \mathrm{ReLU}(U) & \mathrm{ReLU}(-U) \\ \mathrm{ReLU}(-U) & \mathrm{ReLU}(U) \end{pmatrix}. \tag{167}$$

We show below that, assuming weight tying, demanding that the probe stimuli are faithfully reconstructed implies that the matrix U must be semi-orthogonal, *i.e.*,

$$U^\top U = I_d. \tag{168}$$

The tiled representations of $\mathbb{S}^1$ that we considered before are a special case of such encodings (see Appendix C.1).

If the above conditions hold, then the RSM has the general form

$$RSM = \begin{pmatrix} I_d + R & R \\ R & I_d + R \end{pmatrix} \tag{169}$$

where R is a $d \times d$ symmetric matrix with zeros along the diagonal. This matches the structure we observed for $d = 2$, where we saw that

$$RSM = \begin{pmatrix} 1 & \rho & 0 & \rho \\ \rho & 1 & \rho & 0 \\ 0 & \rho & 1 & \rho \\ \rho & 0 & \rho & 1 \end{pmatrix} \tag{170}$$

for a scalar ρ.

The main salient feature of this matrix for $d > 2$ is the fact that its elements obey non-trivial equality relations (relative to the case of $\mathbb{S}^1$, where all non-zero off-diagonal elements of the RSM were equal). For instance, if $d = 3$, this yields an RSM of the form

$$RSM = H^\top H = \begin{pmatrix} 1 & \rho_1 & \rho_2 & 0 & \rho_1 & \rho_2 \\ \rho_1 & 1 & \rho_3 & \rho_1 & 0 & \rho_3 \\ \rho_2 & \rho_3 & 1 & \rho_2 & \rho_3 & 0 \\ 0 & \rho_1 & \rho_2 & 1 & \rho_1 & \rho_2 \\ \rho_1 & 0 & \rho_3 & \rho_1 & 1 & \rho_3 \\ \rho_2 & \rho_3 & 0 & \rho_2 & \rho_3 & 1 \end{pmatrix}. \tag{171}$$

where ρ_1, ρ_2, and ρ_3 are the three distinct non-zero elements of the matrix R. The three functions ρ_1, ρ_2, and ρ_3 are continuous piecewise functions of the three gauge angles that appear in $d = 3$, which can be computed explicitly using Mathematica—though their particular form is not illuminating. We see in Figure 6 that the equality relations between different elements of the RSM implied by (171) are in fact obeyed to high accuracy in experiment. In Figure S9 we show that we observe the corresponding generalized structure (169) empirically in SGD-trained networks for $d = 10$.

[4]Our results extend to larger networks where neurons are duplicated so long as the weights are appropriately normalized. We leave to future work a full investigation of when this form of the RSM applies to larger networks that are equivalent to these small networks thanks to further internal symmetries of the architecture [47, 48].

[5]As long as we assume that the test stimuli are given by a set of d orthonormal vectors along with their negations, we lose no generality in making this choice because the global rotation symmetry implies that we can rotate the basis so that the test stimuli are axis-aligned.

D.1 Structure of the reconstruction

We first show that the matrix U must be semi-orthogonal in order to faithfully reconstruct the probe stimuli S. If we assume weight-tying, we then have the reconstruction

$$\hat{S} = W \operatorname{ReLU}(W^\top S) = (A, -A) \tag{172}$$

where

$$A = U^\top \operatorname{ReLU}(U) - U^\top \operatorname{ReLU}(-U) = U^\top \left[\operatorname{ReLU}(U) - \operatorname{ReLU}(-U) \right]. \tag{173}$$

But, as $\operatorname{ReLU}(x) - \operatorname{ReLU}(-x) = x$ for any $x \in \mathbb{R}$, this simplifies to

$$A = U^\top U. \tag{174}$$

To exactly reconstruct the test stimuli, we should have $\hat{S} = S$ and thus $A = I_d$, which implies that the matrix U must be semi-orthogonal:

$$U^\top U = I_d. \tag{175}$$

Moreover, perfect reconstruction is possible for *any* semi-orthogonal U; choosing one such U corresponds to choosing a gauge.

D.2 Structure of the RSM

We now show that the RSM has the form (169). For this representation, we have the RSM

$$RSM = H^\top H = \begin{pmatrix} Q & R \\ R & Q \end{pmatrix} \tag{176}$$

where we have defined the $d \times d$ blocks

$$Q = \operatorname{ReLU}(U)^\top \operatorname{ReLU}(U) + \operatorname{ReLU}(-U)^\top \operatorname{ReLU}(-U) \tag{177}$$

$$R = \operatorname{ReLU}(U)^\top \operatorname{ReLU}(-U) + \operatorname{ReLU}(-U)^\top \operatorname{ReLU}(U), \tag{178}$$

Noting that R is symmetric, to prove that the RSM takes the form (169) it suffices to show that R has zeros along the diagonal and that $Q = U^\top U + R$.

The fact that R has zeros along the diagonal is easy to see upon expanding in indices:

$$R_{ii} = \sum_{k=1}^{N} [\operatorname{ReLU}(U_{ki}) \operatorname{ReLU}(-U_{ki}) + \operatorname{ReLU}(-U_{ki}) \operatorname{ReLU}(U_{ki})] = 0, \tag{179}$$

as $\operatorname{ReLU}(x) \operatorname{ReLU}(-x) = 0$ for all $x \in \mathbb{R}$.

To show that $Q = U^\top U + R$, we observe that for $x, y \in \mathbb{R}$ we have

$$\begin{aligned} &\operatorname{ReLU}(x) \operatorname{ReLU}(y) + \operatorname{ReLU}(-x) \operatorname{ReLU}(-y) \\ &\quad - \operatorname{ReLU}(x) \operatorname{ReLU}(-y) - \operatorname{ReLU}(-x) \operatorname{ReLU}(y) \\ &\quad = xy. \end{aligned} \tag{180}$$

Applying this identity element-wise, we have

$$\begin{aligned} Q - R &= \operatorname{ReLU}(U)^\top \operatorname{ReLU}(U) + \operatorname{ReLU}(-U)^\top \operatorname{ReLU}(-U) \\ &\quad - \operatorname{ReLU}(U)^\top \operatorname{ReLU}(-U) - \operatorname{ReLU}(-U)^\top \operatorname{ReLU}(U) \qquad (181) \\ &= U^\top U. \qquad (182) \end{aligned}$$

This proves that $Q = U^\top U + R$, and thus that in general we have

$$RSM = \begin{pmatrix} U^\top U + R & R \\ R & U^\top U + R \end{pmatrix}. \tag{183}$$

Note that this applies for any set of weights obeying the reflection-symmetry condition. For a solution satisfying $U^\top U = I_d$, this reduces to (169). As R is a $d \times d$ symmetric matrix with zeros along the diagonal, it has $d(d-1)/2$ independent elements, matching the dimensionality of the gauge group.

E Additional experimental details

E.1 Toy setup

Our toy setup consists of localized RFs on a circle that are created by responses of n ReLU neurons. Specifically, the response of each neuron is $h_i(x) = \mathrm{ReLU}(w_i^\top x - b_i)$, where $w_i = |w_i|(\cos(\theta_i), \sin(\theta_i))$ is the incoming weight defined by θ_i, and b_i is the bias for that neuron. To allow for RFs to have different amplitudes (a_i) and tuning width (δ_i), we set these values accordingly. Given that stimuli lie on a circle, $x = (\cos(\theta), \sin(\theta))$, we have: $h_i(x) = \mathrm{ReLU}(|w_i| \cos(\theta - \theta_i) - b_i)$. The conditions for amplitude and tuning width are $|w_i| - b_i = a_i$ and $|w_i| \cos(\delta_i/2) - b_i = 0$, respectively. Solving for $|w_i|$ and b_i leads to:

$$|w_i| = \frac{a_i}{1 - \cos(\frac{\delta_i}{2})}, \quad b_i = \frac{a_i \cos(\frac{\delta_i}{2})}{1 - \cos(\frac{\delta_i}{2})} \tag{184}$$

Note that in §2 we had $a_i = 1$ and $\delta_i = \pi$, which leads to $b_i = 0$ and $|w_i| = 1$. However, in §4 we used arbitrary amplitudes and tuning widths.

Approximate rescaling of RFs under different tuning widths We remark that in this setting changing the receptive field widths (and number of neurons, to maintain uniform coverage) is nearly, but not exactly, equivalent to re-scaling of the input space. This approximate equivalence follows from the fact that the receptive field shapes change slightly depending on their width. Concretely, consider a receptive field centered at $\theta = 0$, of width δ. Its response as a function of angle is

$$h(\theta, \delta) = \mathrm{ReLU}\left(1 - \frac{1 - \cos(\theta)}{1 - \cos(\delta/2)}\right). \tag{185}$$

For changing the width to be equivalent to a re-scaling of the input, for any new narrower width $\delta' < \delta$ we would like to find a scale factor α such that

$$h(\theta, \delta) \approx h(\alpha\theta, \delta'). \tag{186}$$

This is clearly not feasible globally, as the function

$$\frac{1 - \cos(\theta)}{1 - \cos(\delta/2)} = \left(\frac{\sin(\theta/2)}{\sin(\delta/4)}\right)^2 \tag{187}$$

is not positive-homogeneous in θ. However, we can approximately achieve this locally. Near $\theta = 0$, the desired approximation holds to second order in θ if we take

$$\alpha = \sqrt{\frac{1 - \cos(\delta'/2)}{1 - \cos(\delta/2)}}. \tag{188}$$

This incurs an error near the edges of the receptive field, where the re-scaled receptive field has width

$$\frac{\sin(\delta/4)}{\sin(\delta'/4)}\delta' \tag{189}$$

This is an increasing function of δ', and is equal to δ at $\delta' = \delta$. Therefore, in general with this local approximation there will be some discrepancy between the re-scaled receptive field and the original one. However, this discrepancy is small; if $\delta = \pi$ then the relative width attains a minimal value of $\simeq 0.9003$ as $\delta' \to 0$.

Another way to see the near-equivalence of changing the receptive field width and re-scaling the input is by matching the widths of the RFs. Then, one should choose $\alpha = \delta'/\delta$, which produces a small discrepancy for intermediate values of θ. This can be seen by observing that the ratio of the coefficients of the quadratic terms of the Taylor expansions of $h(\theta, \delta)$ and $h(\delta'\theta/\delta, \delta')$ is

$$\left(\frac{\delta' \sin(\delta/4)}{\delta \sin(\delta'/4)}\right)^2. \tag{190}$$

This ratio is nothing but the square of the ratio of widths we found before; it is again an increasing function of δ' that is close to unity. For instance, with $\delta = \pi$ it is minimized by taking $\delta' \to 0$, where it takes value $\simeq 0.8106$.

Together, these observations give intuition for the approximate collapse of curves we saw in Figure 3: the RSM variability for different tuning widths and different numbers of neurons should approximately depend only on the product of the tuning width and the number of neurons because changing the width is *nearly* equivalent to expanding the input space.

E.2 Neural network simulations

Here we provide experimental details regarding the simulations provided throughout the paper. Neural networks consisted of a two-layer autoencoder with input dimension d, hidden-layer with ReLU activation consisting of n neurons, and an output layer that was a reconstruction of the input ($y = x$). Networks were trained using a vanilla SGD with a fixed learning rate (η), a weight decay (γ) and varying batch size (b). In some simulations, an L_1 penalty on the activations of the hidden layer was imposed. Training was performed on PyTorch with NVIDIA GeForce RTX 2080 Ti GPU. Specifics parameters are mentioned for each simulation below.

E.2.1 Simulations with Gaussian data

Data were drawn in an online way from normal d-dimensional standard Gaussian distribution. Prior to the online training, a warm-up pretraining was performed with no weight decay and batch size of 128 (this corresponds to time 0 on the axes of the training plots). The plots of RSM values over time were created by first saving snapshots of the model at intervals of 100 time steps, and then applying a moving average filter with the window size of 5. The specific parameters for each figure are as follows:

Figure S6: $d = 2$, $n = 4$, $\eta = 0.1$, $\gamma = 0.1$, $b = 1$.

Figure 4: $d = 2$, $n = 15$, $\eta = 0.15$, $\gamma = 0.1$, $b = 1$.

Figure 5: $d = 2$, $n = 15$, $\eta = 0.1$, $\gamma = 0.1$, $b = 100$, $\lambda_1 = 0.001$ (L1-penalty coefficient).

Figure 6: $d = 3$, $n = 6$, $\eta = 0.1$, $\gamma = 0.1$, $b = 1$.

Figure S7: $d = 2$, $n = 4$, $\eta = 0.1$, $\gamma = 0.1$, $b = 1$.

Figure S8: $d = 2$, $n = 15$, $\eta = 0.1$, $\gamma = 0.1$, $b = 100$.

Figure S9: $d = 10$, $n = 20$, $\eta = 0.075$, $\gamma = 0.05$, $b = 1$.

E.2.2 Simulations with rotated image data (Figures 7 and S11)

As mentioned in §7 of the main text, here the data consists of rotated versions of a digit from the Kuzushiji-MNIST dataset [22]. Specifically, we take one digit with dimensions 28×28 and create 180 rotated versions of it covering the full circle. The baseline image was a digit ($index = 1$) from Kuzushiji-49 training dataset, and was accessed from `https://github.com/rois-codh/kmnist`, where it is available under a CC BY-SA 4.0 License.[6]

The image data were flattened and fed into a two-layer autoencoder with ReLU nonlinearity and trainable bias. The input/output and the hidden layer dimensions were 784 and 32 respectively. Each model was trained using SGD with batch size $b = 24$, learning rate $\eta = 0.01$, weight decay $\gamma = 0.005$, and L_1 penalty coefficient 2×10^{-5} on hidden layer activations. Additionally, a small Gaussian synaptic noise was added to the weights for regularization (noise variance $\eta b \sigma^2$ for $\sigma = 0.02$). The training was continued for 500 epochs which is long after the training loss stabilized.

Overall, these parameters allowed us to create approximately localized RFs, although this is not the only way to obtain such receptive fields. Finally, 1000 runs of the above training was performed with random initializations to achieve multiple instances of RFs. These simulations led to different number of RFs, as shown in the histogram in Fig. S11. Nevertheless, the findings were consistent across all numbers. The results in Fig. 7 of the main text correspond to models with $n = 15$ RFs. Finally, to infer φ, a max projection of active RFs was performed to find the RF envelopes, and the phase of the dominant frequency of the envelope was chosen as the gauge variable for that run. This is illustrated in Fig. S10.

E.3 Pretrained vision models (Figure S13)

We further studied representations of the rotated image data in three exemplary pretrained vision models. As convolutional baselines, we used ResNet-18, a standard residual CNN with approximately 11.7M parameters [23], and ConvNeXt-Tiny, a more modern convolutional architecture with

[6]Compared to MNIST digits, this image contained fewer intrinsic symmetries, which led to a smaller degree of interference with the latent symmetry. For example, using the numeral "0" would include a four-fold intrinsic symmetry of the digit.

roughly 28.6M parameters [24]. For both CNNs, we analyzed the final-stage, global-average-pooled feature representations. As a transformer-based model, we used DINOv2-S/14-reg, which is a self-supervised Vision Transformer with 12 transformer blocks, 384-dimensional embeddings, 14×14 patches, and register tokens, with roughly 21M parameters; representations were taken from the final-block class token (`block_11`) [25]. The inputs are 360 rotated versions of the baseline image in Fig. 7a (from the Kuzushiji-MNIST dataset), color-coded by their rotation angle. ResNet-18 and ConvNeXt-Tiny pretrained weights were obtained through `torchvision.models` using the default weight configurations, and pretrained DINOv2-S/14-reg weights were obtained from the Torch Hub repository.

F Additional figures

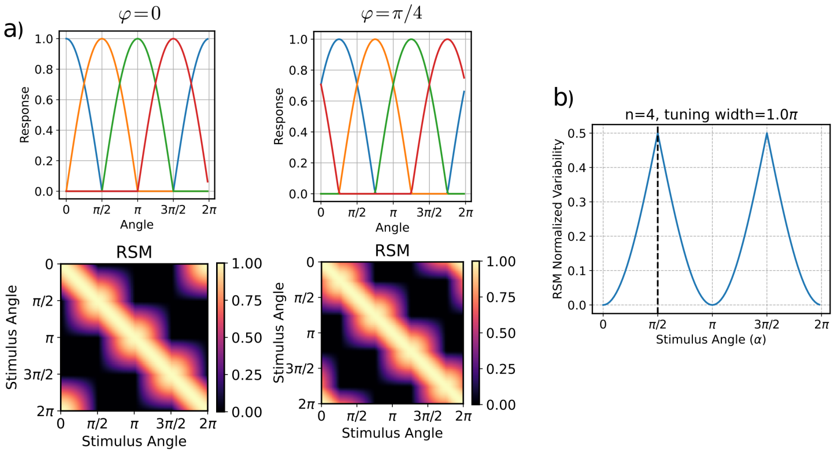

Figure S1: Stimulus-dependency of RSM and its dependence on φ. a) Examples of full-RSM matrices for two values of $\varphi = 0$ and $\varphi = \pi/4$. b) Dependency of normalized RSM variability (Δ) on the stimulus angle. Here, the RSM are calculated between $s_1 = (1, 0)^\top$ and $s_\alpha = (\cos\alpha, \sin\alpha)^\top$. All cases are for $n = 4$ with tuning width of π, which is the same as the toy model in Fig. 1.

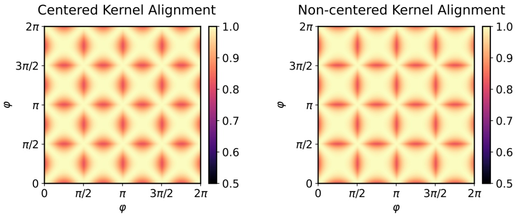

Figure S2: Kernel alignment measures inherit gauge-dependency from RSM. (Left) Centered Kernel Alignment (CKA) and (Right) non-centered kernel alignment between pairs of RSM matrices at different values of gauge variable. The values are calculated based on the toy setup of Figure 1 with $n = 4$ neurons and four trial stimuli. See also Appendix A.4.

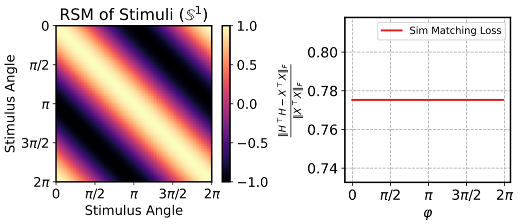

Figure S3: Gauge-dependency of RSM does not affect similarity matching loss. (left) Stimulus RSM $X^\top X$ on a ring ($\mathbb{S}^1$). (right) Similarity matching loss $\frac{\|H^\top H - X^\top X\|_F}{\|X^\top X\|_F}$ as a function of gauge variable (setup is similar to Fig. S1, and RSM is calculated with 100 trial stimuli). We prove that this holds more generally in Appendix B.3.

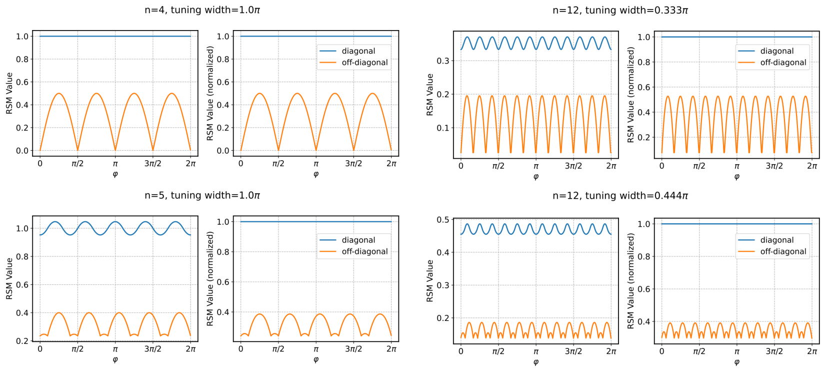

Figure S4: Additional examples of φ-dependency of RSMs for different numbers of RF and tuning widths. In all cases, the off-diagonal RSM is calculated for stimulus $s_1 = (1, 0)^\top$ and $s_\alpha = (\cos\alpha, \sin\alpha)^\top$, for α half of the tuning width.

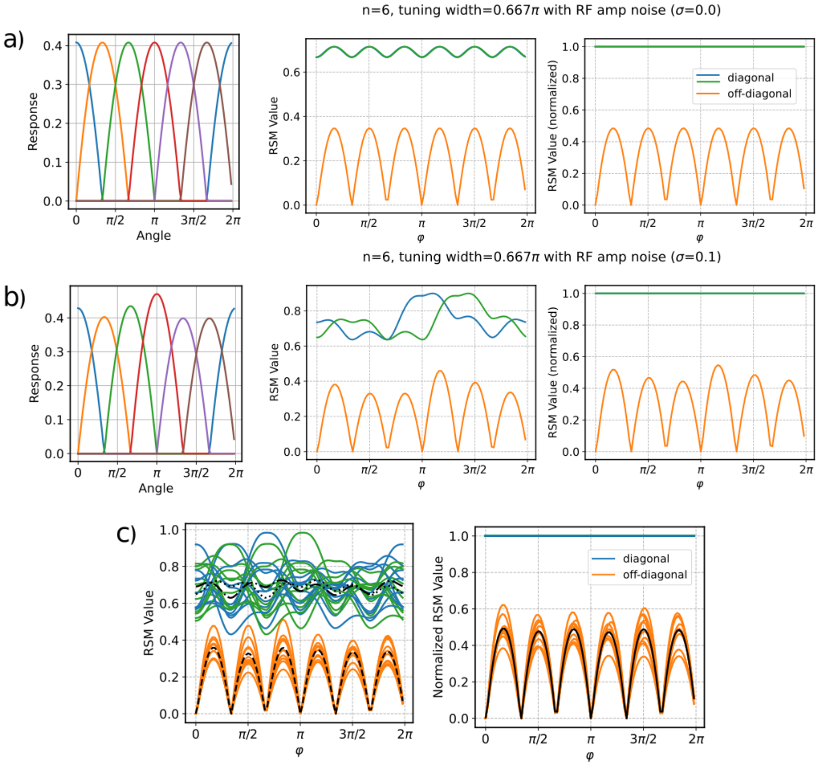

Figure S5: Additional results on RSM variability in neural codes with RF amplitude noise. Amplitude noise is added to existing RF as Gaussian noise with standard deviation $std = \sigma \times amp$, where amp is the baseline amplitude. a) $\sigma = 0$. b) $\sigma = 0.1$. c) Average curves for 100 realizations of amplitude noise. In all cases, the blue and green curves correspond to diagonal elements corresponding to stimuli $s_1 = (1, 0)^\top$ and $s_\alpha = (\cos\alpha, \sin\alpha)^\top$ for α half of the tuning width, respectively. Similarly, the orange off-diagonal curves show RSM entries between s_1 and s_α.

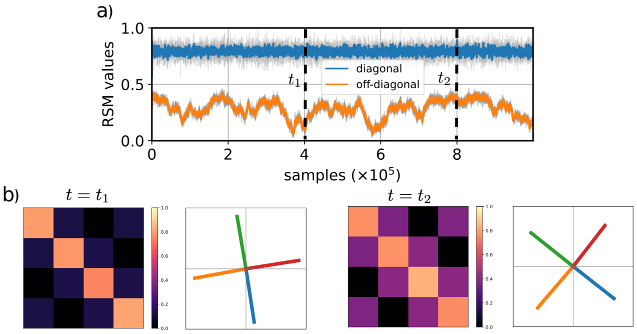

Figure S6: Continual training under SGD leads to a variable RSM. Input is two-dimensional and there are $n = 4$ ReLU neurons. a) Values of RSM over time. b) At two time snapshots of training, the RSM matrices (left), and the corresponding weight vectors (right) are shown.

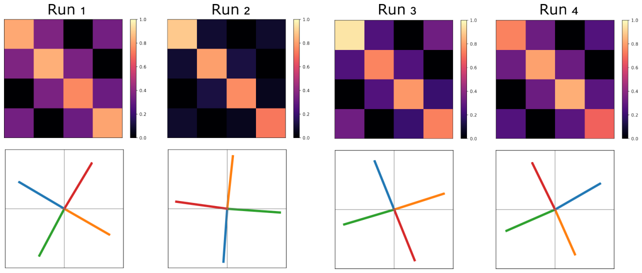

Figure S7: RSM plots (top) and neurons' weight vectors (bottom) for four different runs and $n = 4$ neurons. Each simulation is run with 5×10^4 samples seen (batch size of 1).

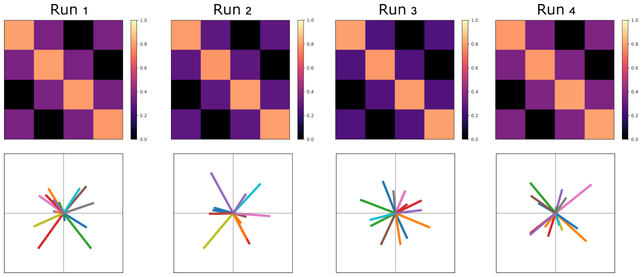

Figure S8: RSM plots (top) and neurons' weight vectors (bottom) for four different runs and $n = 15$ neurons. The batch size is large, leading to a relatively low SGD-noise regime where, unlike Fig. 4 in the main text, neurons' weights do not collapse. Each simulation is run with 5×10^4 samples seen (batch size of 100).

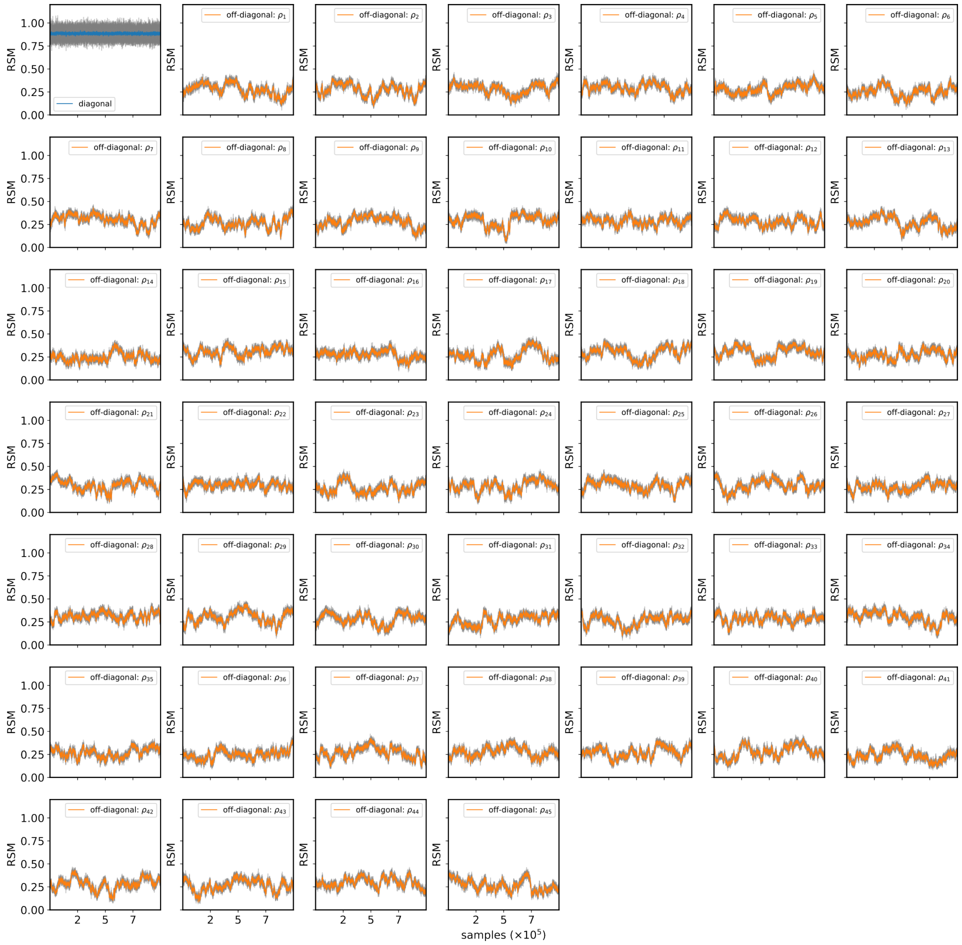

Figure S9: RSM values over time for a network with input dimension $d = 10$ and $n = 20$ neurons. Network is trained with SGD and batch size of one. Based on the predictions in Appendix D, the non-zero off-diagonal elements are placed into $d(d-1)/2 = 45$ groups. Each group contains 4 RSM values which, as predicted, are highly correlated (gray curves: members of the group, orange: group mean).

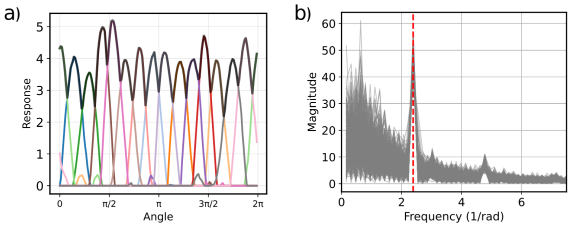

Figure S10: Inference of the gauge variable φ from RF profiles of trained networks. a) An example of a run with $n = 15$ RFs. Black line shows the envelope of the RFs found by max projection. b) Frequency spectrum of the RF envelopes for multiple runs. For each run, φ is inferred by finding the phase at the dominant frequency at $n/2\pi$ (shown by red dashed line).

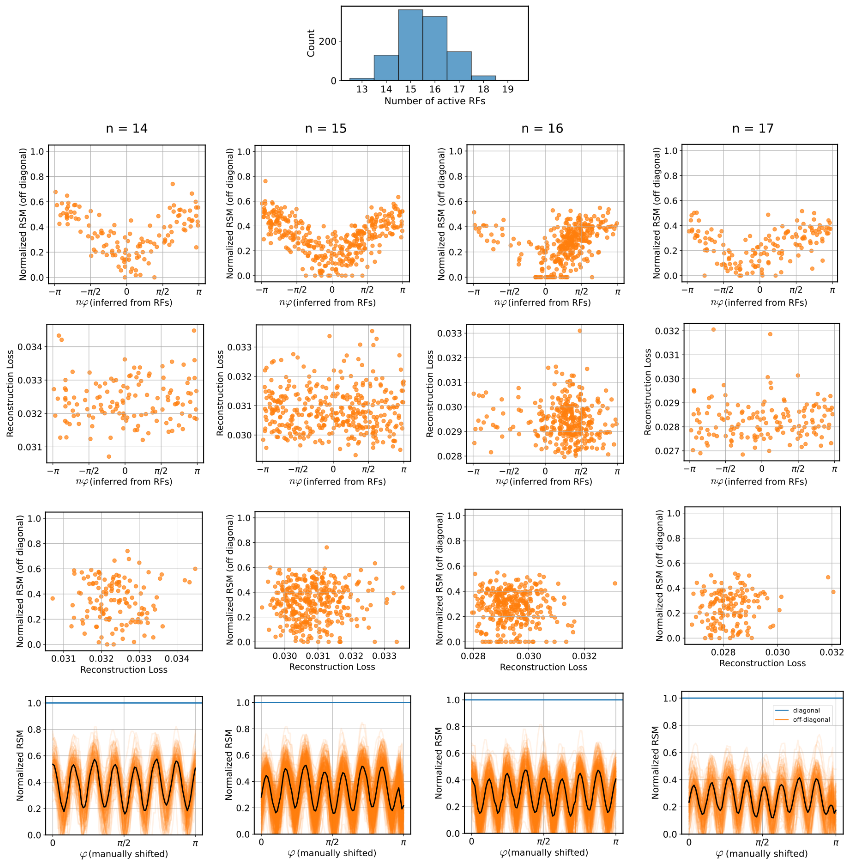

Figure S11: Additional results on RSM variability in real-world data (see Fig. 7 and the main text for details of the plots). 1000 realizations of the autoencoder were trained on rotated versions of a given image. The histogram on top shows the distribution of the number of RFs across runs. For each number, the associated plots of RSM variability are shown in a column. The φ-dependency of RSMs is evident irrespective of the number of RFs. In all plots, the orange data correspond to the off-diagonal entry of the RSM and are calculated for two stimuli that are $\alpha = 25^{\cdot}$ apart.

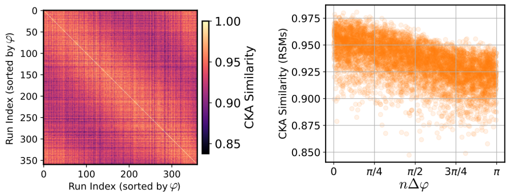

Figure S12: Additional results on CKA analysis on autoencoding image data with latent symmetry in Section 7. The analysis is similar to panels g and h in Figure 7, but CKA similarity is measured from non-normalized RSMs. (Left) CKA similarity matrix calculated between RSMs of different runs. The runs are sorted based on the inferred φ. (Right) Scatter of CKA similarity as a function of gauge difference (each point represents a pair of runs, and for visualization purposes only $1/10$ of the data are shown; Pearson's $r = -0.56, p < 0.001$).

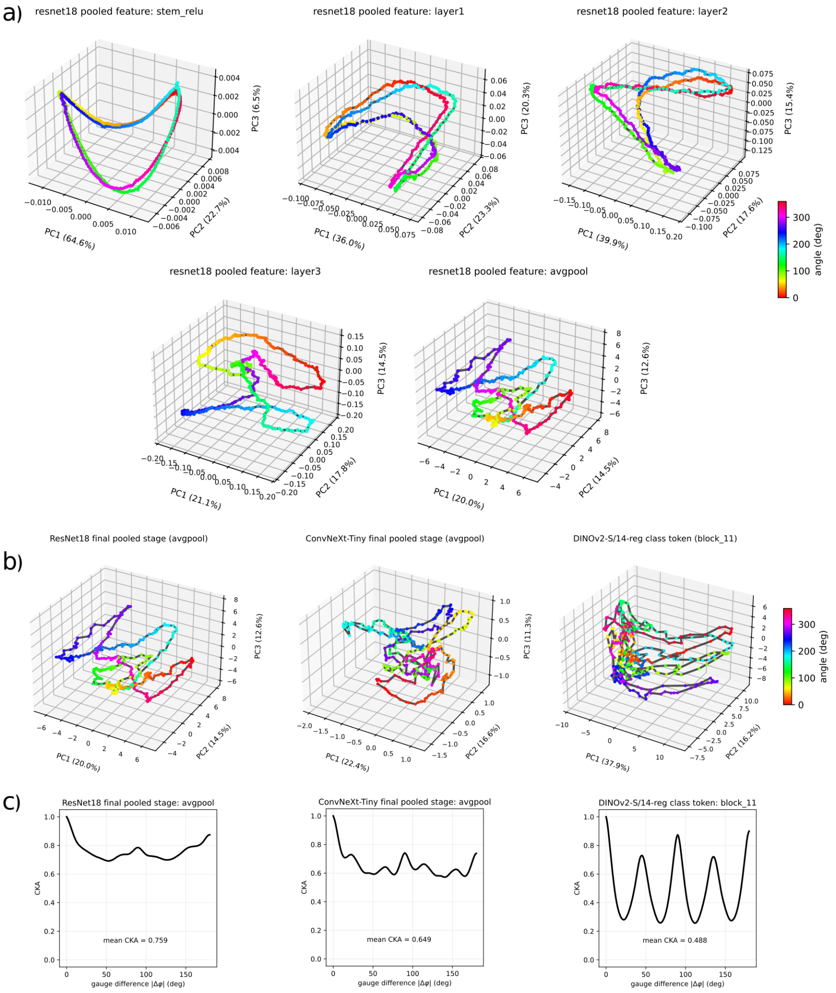

Figure S13: Representations of rotated images in pretrained vision models. a) 3D PCA embeddings across layers of ResNet-18. Color denotes rotation angle. b) Embeddings in the final layers of three exemplary pretrained models: (left) ResNet-18 [23], (middle) ConvNeXt-Tiny [24], and (right) DINOv2-S/14-reg [25]. The first two models are convolution-based, and the last model is transformer-based. c) CKA similarity as a function of gauge difference for the three models in (b). Note that here, different gauge values were created manually by offsetting the RSM according to the starting angle. This corresponds to circularly shifting the rows and columns of RSM simultaneously.
